# Autophagic degradation of caveolin-1 promotes liver sinusoidal endothelial cells defenestration

**DOI:** 10.1038/s41419-018-0567-0

**Published:** 2018-05-14

**Authors:** Xiaoying Luo, Dan Wang, Xintao Zhu, Guozhen Wang, Yuehua You, Zuowei Ning, Yang Li, Siyi Jin, Yun Huang, Ye Hu, Tingting Chen, Ying Meng, Xu Li

**Affiliations:** 1 State Key Laboratory of Organ Failure Research, Guangdong Provincial Key Laboratory of Viral Hepatitis Research, Department of Infectious Diseases, Nanfang Hospital, Southern Medical University, Guangzhou, China; 2 Guangdong Provincial Key Laboratory of Gastroenterology, Department of Gastroenterology, Nanfang Hospital, Southern Medical University, Guangzhou, China; 3 Department of Stomatology, People’s hospital of Longhua, Shenzhen, Guangdong China; 4 Department of Respiratory Diseases, Nanfang Hospital, Southern Medical University, Guangzhou, China

## Abstract

Autophagy, interacting with actin cytoskeleton and the NO-dependent pathway, may affect the phenotype and function of endothelial cells. Moreover, caveolin-1 (Cav-1), as a structure protein in liver sinusoidal endothelial cells (LSECs), is closely related to autophagy. Hence, we aim to explore the role of autophagic degradation of Cav-1 in LSECs defenestration. In vivo, we found the increase of autophagy in liver sinusoidal endothelium in human fibrotic liver. Furthermore, autophagy, degradation of Cav-1, and actin filament (F-actin) remodeling were triggered during the process of CCl4-induced LSECs defenestration; in contrast, autophagy inhibitor 3MA diminished the degradation of Cav-1 to maintain fenestrae and relieve CCl4-induced fibrosis. In vitro, during LSECs defenestration, the NO-dependent pathway was down-regulated through the reduction of the PI3K–AKT–MTOR pathway and initiation of autophagic degradation of Cav-1; while, these effects were aggravated by starvation. However, VEGF inhibited autophagic degradation of Cav-1 and F-actin remodeling to maintain LSECs fenestrae via activating the PI3K–AKT–MTOR pathway. Additionally, inhibiting autophagy, such as 3MA, bafilomycin, or ATG5-siRNA, could attenuate the depletion of Cav-1 and F-actin remodeling to maintain LSECs fenestrae and improve the NO-dependent pathway; in turn, eNOS-siRNA and L-NAME, for blocking the NO-dependent pathway, could elevate autophagic degradation of Cav-1 to aggravate defenestration. Finally, overexpressed Cav-1 rescued rapamycin-induced autophagic degradation of Cav-1 to maintain LSECs fenestrae; whereas knockdown of Cav-1 facilitated defenestration due to the activation of the AMPK-dependent autophagy. Consequently, autophagic degradation of Cav-1 promotes LSECs defenestration via inhibiting the NO-dependent pathway and F-actin remodeling.

## Introduction

The liver sinusoidal endothelial cells (LSECs) are characterized with possession of fenestrae, whose disappearance is implicated in liver fibrogenesis and cirrhosis^1,2^. To explore the underlying mechanism and the therapeutic target of chronic liver diseases, scientists concentrate on the promoter of the dysregulation of LSECs phenotype^3^. It is confirmed that caveolin-1 (Cav-1) and actin cytoskeleton (such as F-actin), which are closely affiliated with LSECs fenestration, could regulate the contraction and dilatation of the fenestrae^4–6^. In other words, the changes and migration of Cav-1 or F-actin remodeling might influence LSECs phenotype.

Autophagy is a process that regulates cellular homeostasis and eliminates the damaged proteins or organelles^7,8^. In liver pathological conditions, autophagy plays different roles in phenotype and function of intra-hepatic cells. For example, autophagy protects hepatocytes and macrophages from damages to improve liver injury or fibrosis^9,10^. However, serious autophagy activates hepatic stellate cells (HSCs) and biliary epithelial cells to aggravate liver fibrogenesis^11–13^. However, the literature about the effects of autophagy on LSECs phenotype and function is controversial. In ischemia/reperfusion (I/R)-induced acute liver injury, statins improve the hepatic endothelial microvascular function through the inhibition of Rac1, which consequently activates autophagy and increments the expression of KLF2^14^. In liver fibrosis, our previous studies for the first time revealed that Cav-1-related autophagy, initiated by aldosterone-induced oxidation, promotes LSECs defenestration^15^. However, the mechanism underlying the effects of autophagy on the regulation of LSECs defenestration is still unclear.

Autophagy displays links with actin cytoskeleton and Cav-1 in LSECs. Actin cytoskeleton, an important part of fenestrae for controlling its contraction, could regulate autophagosome maturation, and in turn, autophagy facilitates F-actin remodeling^16^, implying that autophagy regulates F-actin remodeling to promote LSECs shrinking. Cav-1, a crucial protein around the fenestrae and on vesicles, participates in autophagy. On the one hand, Cav-1 is responsible for energy generation: Cav-1 upregulation increases glucose uptake and ATP production by stimulating the glucose transporter 3 (GLUT3). In contrast, the depletion of Cav-1 inhibited glucose uptake and ATP generation, and triggered autophagy via AMPK signaling in colorectal tumor cells^17,18^. On the other hand, Cav-1 connects with the ATG12-ATG5 system to suppress autophagy^19^.

Hence, Cav-1 and autophagy, interacting with each other, may participate in LSECs defenestration via promoting F-actin remodeling. To further investigate the interaction of Cav-1 and autophagy, as well as their roles in LSECs defenestration, we assessed the response of primary rat LSECs to vascular endothelial growth factor (VEGF) or starvation, which is known to maintain LSECs fenestrae or promote LSECs defenestration^20,21^.

## Results

### Cav-1 is closely related to autophagy in liver sinusoidal endothelium in human liver fibrosis

**Fig. 1:**
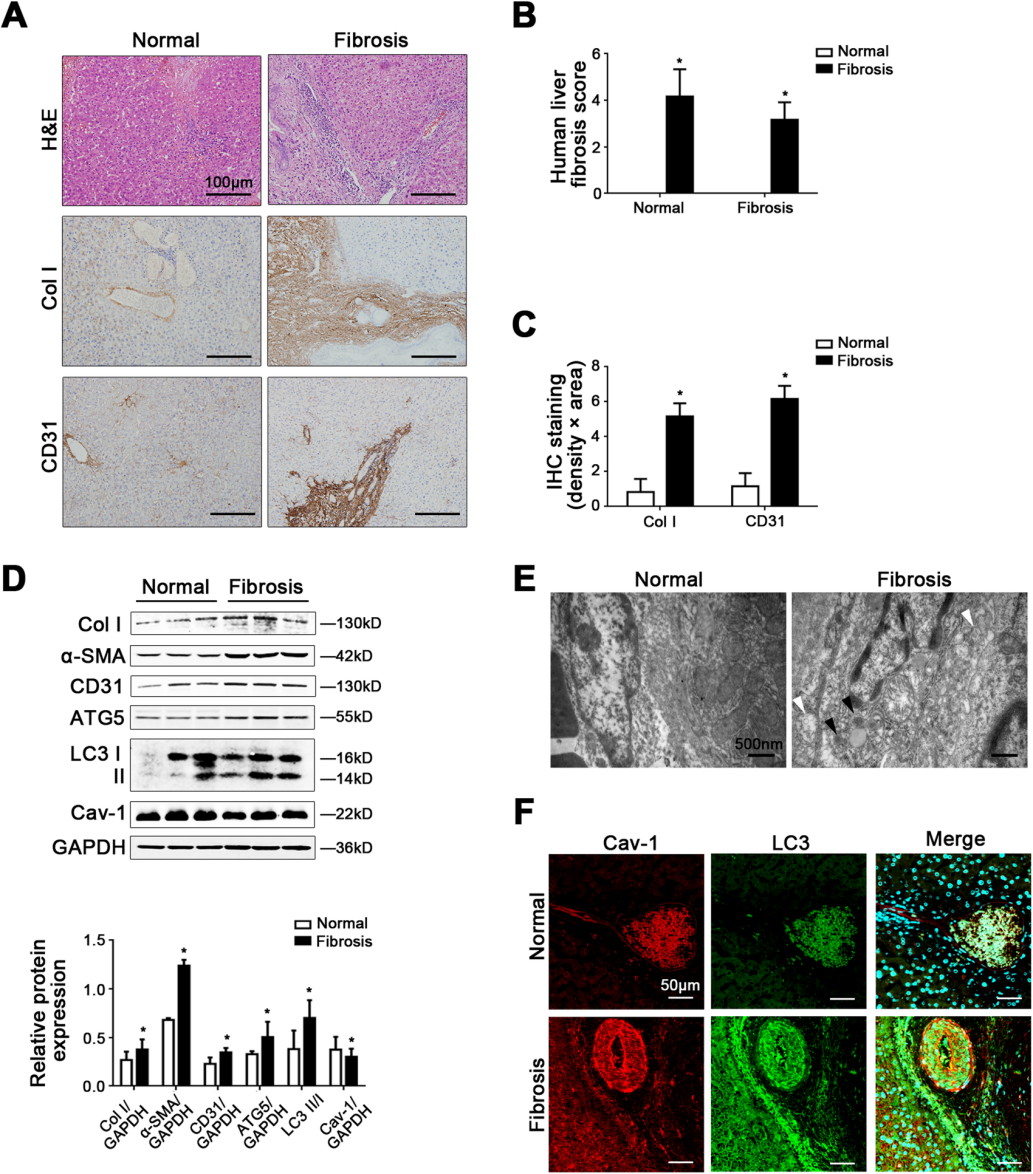
Cav-1 is closely related to autophagy in liver sinusoidal endothelium in human liver fibrosis. **a** The H&E and the immunohistochemical staining (IHC) of Col I and CD31 in liver biopsy specimens (scale bar: 100 μm). **b** The quantified analysis of liver fibrosis with ISHAK and Metavir score. *P < 0.05 versus the normal group. **c** The area density of IHC of Col I and CD31. *P < 0.05 versus the normal group. **d** Protein expression of Col I, α-SMA, CD31, ATG5, LC3 II/I, and Cav-1 in the liver tissue. The relative protein expression is quantified in the graph, down. *P < 0.05 versus the normal group. **e** Autophagosomes structures (denoted by white triangles) and autolysosomes structures (denoted by black triangles) of the liver sinusoidal endothelium in human liver tissue shown in a high-magnification TEM (scale bar: 500 nm). **f** The co-localization of LC3 with Cav-1 by immunofluorescence (scale bar: 50 μm)

Compared with the normal group, the fibrosis level, and the protein levels of collagen I (Col I), α-smooth muscle actin (α-SMA), and CD31 of human liver fibrotic tissue were higher (Fig. 1a–d). Moreover, the protein expression of ATG5 and LC3 II/I, as well as the data of transmission electron microscopy (TEM), showed that autophagy was activated in liver sinusoidal endothelium in human liver fibrosis (Fig. 1d, e). Besides, in human liver fibrotic tissue, Cav-1 protein expression decreased, but more Cav-1 co-localized with LC3 in liver sinusoidal endothelium (Fig. 1d, f). Hence, these implied that Cav-1-associated autophagy might occur in capillarized liver sinusoidal endothelium in liver fibrosis.

### Inhibiting autophagy attenuates CCl4-induced LSECs defenestration and fibrosis

The data of scanning electron microscopy (SEM) showed that before CCl4-induced liver fibrogenesis, LSECs defenestration occurred on the sixth day. Meanwhile, the molecules of the NO-dependent pathway (namely the NO/eNOS/sGC/cGMP/PKG/VASP pathway), such as eNOS and VASP, were downregulated; while CD31 was highly expressed in primary LSECs, isolated from the CCl4 model, from Day 6 till Day 28 (Supplementary Figure 1A, B). Interestingly, during the first stage of CCl4-induced liver fibrosis, there was a time-dependent elevation of LC3 II/I protein expression, along with the decrease of Cav-1 protein level in LSECs (Supplementary Figure 1B). Furthermore, the immunofluorescence showed that LC3 was highly expressed in vWF-positive liver sinusoidal endothelium on the sixth day (Supplementary Figure 1C). Hence, these results confirmed that during the initiation of fibrosis in vivo, CCl4 induced defenestration, a pro-fibrotic phenotype transition of LSECs, through serious autophagy, as well as the downregulation of Cav-1 and the NO-dependent pathway.

**Fig. 2:**
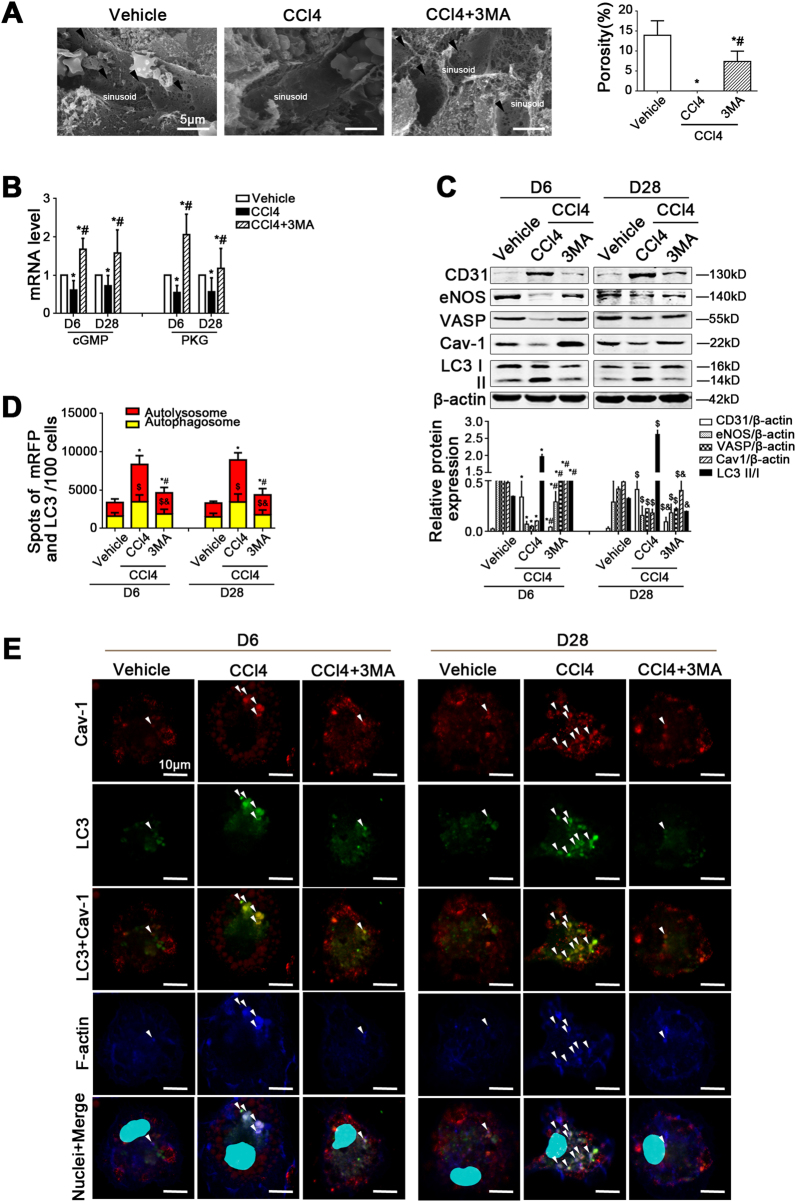
Inhibiting autophagy attenuates CCl4-induced LSECs defenestration. **a** Magnification SEM of liver sinusoidal endothelium in rat liver on Day 6 showing the fenestrae (scale bar: 5 μm), and quantification of porosity in LSECs of CCl4 rat models, right. The black arrows indicate LSECs fenestrae structures. *P < 0.05 versus the vehicle group; ^#^*P* < 0.05 versus the CCl4 group. **b** Real-time PCR analysis of cGMP and PKG mRNA levels in primary LSECs isolated from CCl4 rat models. *P < 0.05 versus the vehicle group on Day 6 or Day 28; ^#^*P* < 0.05 versus the CCl4 group on Day 6 or Day 28. **c** Representative immunoblots of CD31, eNOS, VASP, Cav-1, and LC3 II/I in primary LSECs isolated from CCl4 rat models. *P < 0.05 versus the vehicle group on Day 18; ^#^*P* < 0.05 versus the CCl4 group on Day 18; ^$^*P* < 0.05 versus the vehicle group on Day 28; ^&^*P* < 0.05 versus the CCl4 group on Day 28. **d** Monitoring of LSECs autophagic flux using a dual fluorescence mRFP-GFP-LC3 marker. Red or yellow represents autolysosomes or autophagosomes, respectively, visualized by confocal microscopy. Quantification of autophagic flux (%) in 100 cells was analyzed with Image J software. *P < 0.05 versus autolysosomes of the vehicle group on Day 6 or Day 28; ^#^*P* < 0.05 versus autolysosomes of the CCl4 group on Day 6 or Day 28; ^$^*P* < 0.05 versus autophagosomes the vehicle group on Day 6 or Day 28; ^&^*P* < 0.05 versus autophagosomes of the CCl4 group on Day 6 or Day 28. **e** The co-localization of LC3 (green) with Cav-1 (red) and F-actin (blue) in LSECs of the three groups (vehicle, CCl4, CCl4+3MA), shown by immunofluorescence. Scale bar: 10 μm; *n* = 6 per group

However, the data of SEM, the cGMP and PKG mRNA levels, the autophagic flux, as well as the protein levels of eNOS, VASP, Cav-1, and LC3 II/I showed that LSECs fenestrae were maintained by 3MA on the sixth day due to the inhibition of autophagy, concomitant with the upregulation of Cav-1 expression and the NO-dependent pathway (Fig. 2a–d). In addition, the immunofluorescence showed that compared with the control group, the co-localization of LC3 with Cav-1 and F-actin increased in the perinuclear area of LSECs in the CCl4 group. In contrast, in the 3MA-treated group, less LC3 co-localized with Cav-1 and F-actin; moreover, Cav-1 and F-actin were distributed uniformly around the cell membrane (Fig. 2e), impliying that the degradation of Cav-1 and F-actin remodeling was triggered due to serious autophagy during CCl4-induced LSECs defenestration; however, these effects were rescued by autophagy inhibitor 3MA.

**Fig. 3:**
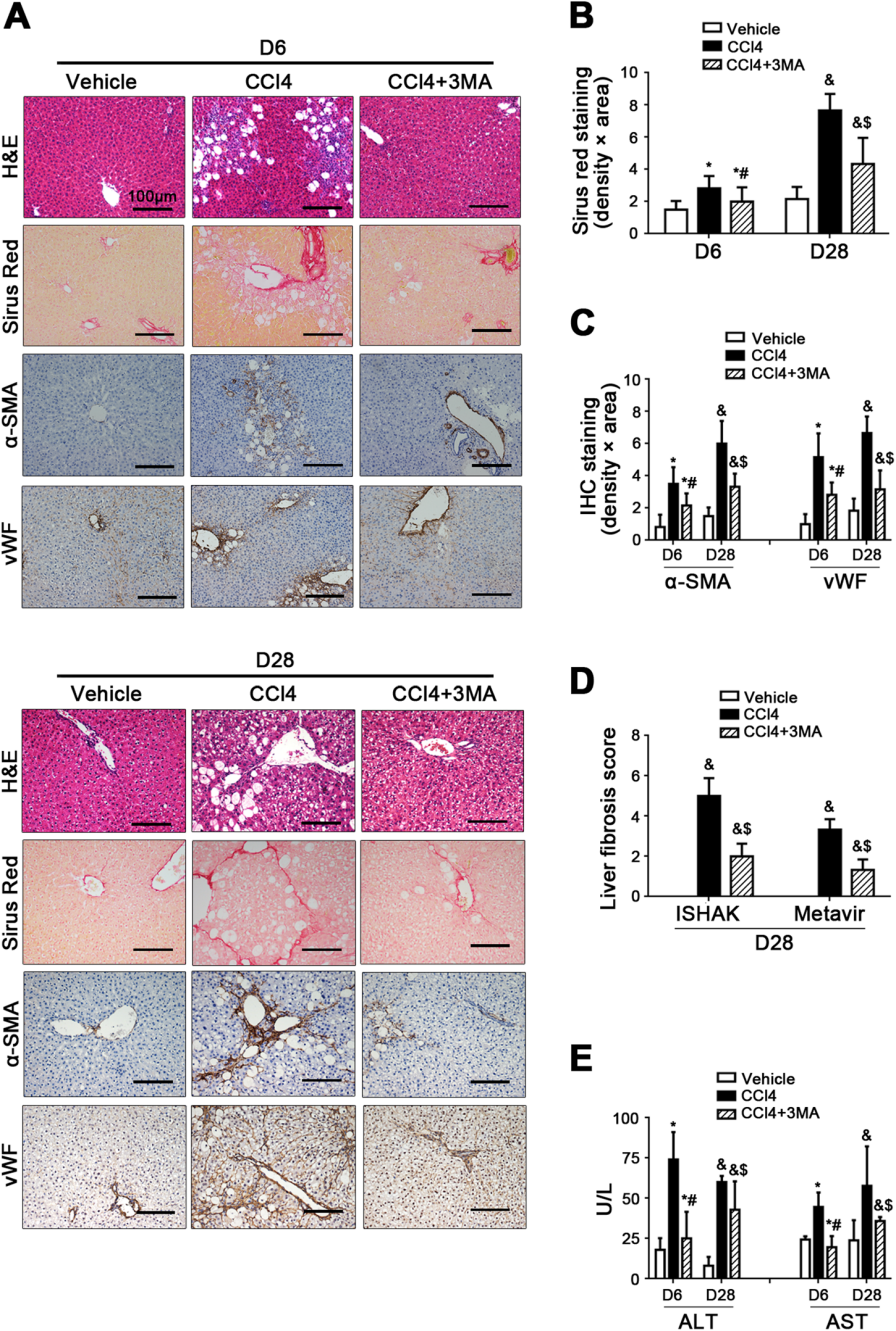
Inhibiting autophagy relieves CCl4-induced liver fibrosis. **a** The H&E, Sirius Red, and IHC for α-SMA and vWF staining of liver tissue on Day 6 and Day 28 (scale bar: 100 μm). **b** The area density of Sirus Red staining of liver tissue on Day 6 and Day 28. *P < 0.05 versus the vehicle group on Day 6; #*P* < 0.05 versus the CCl4 group on Day 6; ^&^*P* < 0.05 versus the vehicle group on Day 28; ^$^*P* < 0.05 versus the CCl4 group on Day 28. **c** Semiquantitative score of IHC staining for α-SMA and vWF staining of liver tissue on Day 6 and Day 28. *P < 0.05 versus the vehicle group on Day 6; ^#^*P* < 0.05 versus the CCl4 group on Day 6; ^&^*P* < 0.05 versus the vehicle group on Day 28; ^$^*P* < 0.05 versus the CCl4 group on Day 28. **d** The quantification of liver fibrosis with ISHAK and Metavir score on Day 28. ^&^*P* < 0.05 versus the vehicle group; ^$^*P* < 0.05 versus the CCl4 group. **e** The ALT and AST content on Day 6 and Day 28. *P < 0.05 versus the vehicle group on Day 6; #*P* < 0.05 versus the CCl4 group on Day 6; ^&^*P* < 0.05 versus the vehicle group on Day 28; ^$^*P* < 0.05 versus the CCl4 group on Day 28

Furthermore, inhibiting autophagy could alleviate CCl4-induced liver fibrosis and the contents of serum glutamic pyruvic transaminase (ALT) and glutamic oxaloacetic transaminase (AST) (Fig. 3).

Hence, these data suggested that serious autophagy initiated the degradation of Cav-1, along with F-actin remodeling and the downregulation of the NO-dependent pathway, during CCl4-induced LSECs defenestration in the early stage of liver fibrosis. Autophagy inhibitor 3MA could attenuate these effects to maintain LSECs fenestrae and improve liver fibrosis.

### Autophagic degradation of Cav-1 emerged during the progression of LSECs defenestration

**Fig. 4:**
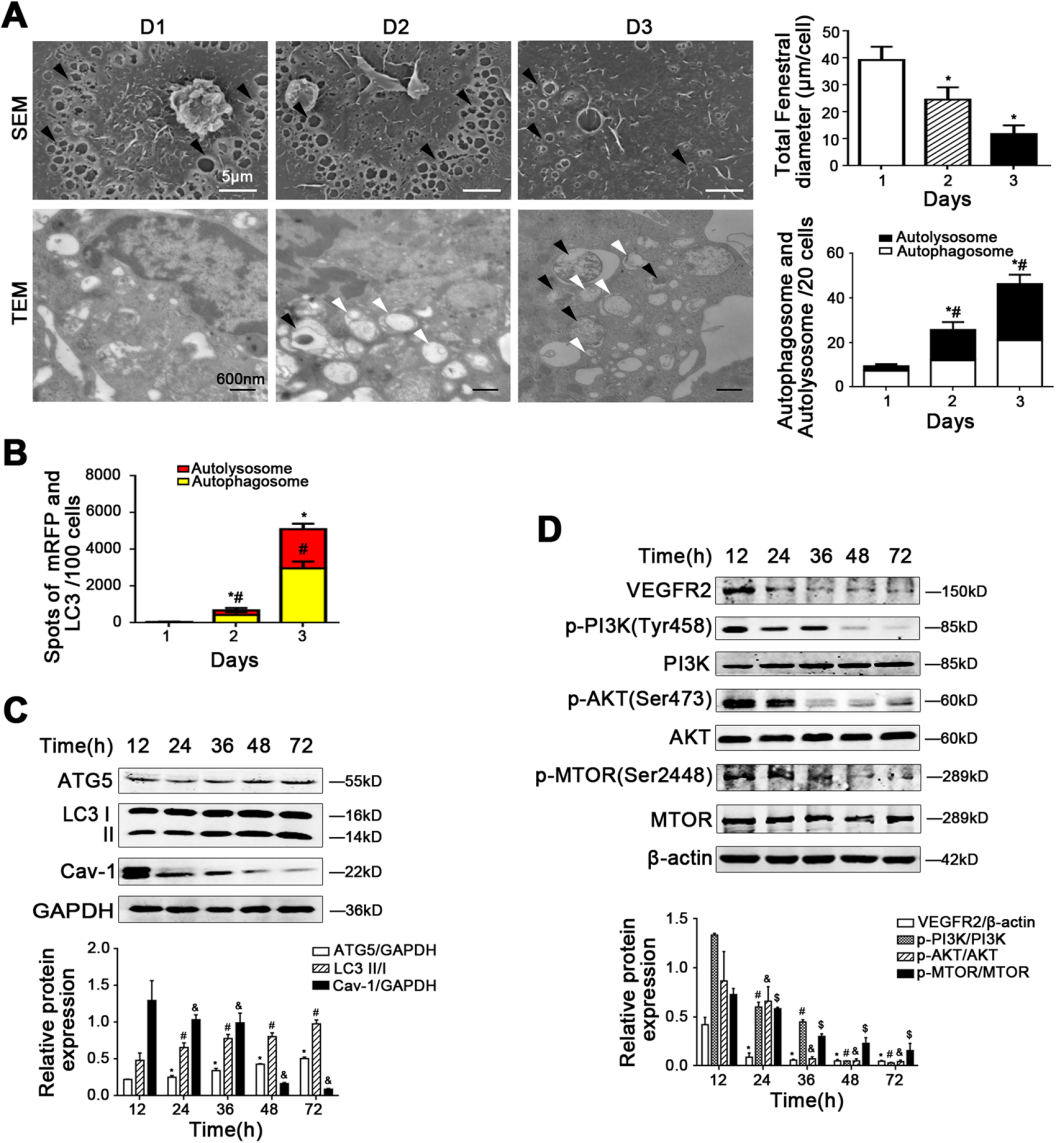
Autophagy obviously rises with the degradation of Cav-1 during the progression of LSECs defenestration. Primary rats LSECs, isolated from normal rats, were cultured for 3 days in vitro. **a** (Up) Magnification of SEM of LSECs on Day 1, Day 2, and Day 3, revealing the fenestrae structures (scale bar: 5 μm), and quantification of the total fenestral diameter, right. The black triangles indicate LSECs fenestrae structures. *P < 0.05 versus the Day 1 group. (Down) Autophagosomes structures (denoted by white triangles) and autolysosomes structures (denoted by black triangles) in LSECs shown in a high-magnification TEM and bar graphs showing counts of autophagosomes and autolysosomes, right. *P < 0.05 versus the autophagosomes of the Day 1 group; ^#^*P* < 0.05 versus the autolysosomes of the Day 1 group. **b** Red or yellow represents autolysosomes or autophagosomes, respectively, visualized by confocal microscopy. Quantification of autophagic flux (%) in 100 cells was analyzed. *P < 0.05 versus the autolysosomes of the Day 1 group; ^#^*P* < 0.05 versus the autophagosomes of the Day 1 group. **c** Protein levels of ATG5, LC3 II/I, and Cav-1 in primary LSECs analyzed by western blot. The relative protein expression is quantified in the graph, down. *P < 0.05 versus ATG5/GAPDH of the 12 h group; ^#^*P* < 0.05 versus LC3 II/I of the 12 h group; ^&^*P* < 0.05 versus Cav-1/GAPDH of the 12 h group. **d** Protein levels of VEGFR2, p-PI3K (Tyr458), PI3K, p-AKT (Ser473), AKT, p-MTOR (Ser2448), and MTOR analyzed by western blot. The relative protein expression is quantified in the graph, down. *P < 0.05 versus VEGFR2/β-actin of the 12 h group; ^#^*P* < 0.05 versus p-PI3K/PI3K of the 12 h group; ^&^*P* < 0.05 versus p-AKT/AKT of the 12 h group; ^$^*P* < 0.05 versus p-MTOR/MTOR of the 12 h group

The fenestrae of primary LSECs isolated from normal rats shrank rapidly from Day 1 to Day 3 during culturing without growth factors in vitro, along with the downregulation molecules of the NO-dependent pathway, such as sGC, cGMP, PKG, eNOS, and VASP (Fig. 4a, Supplementary Figure 2A-C). Interestingly, the data of TEM, the autophagic flux, the protein levels of ATG5, LC3 II/I, and Cav-1, as well as the co-localization of Cav-1 and LC3, in LSECs showed that fenestrae were shrinking, along with the augment of autophagy and the degradation of Cav-1 (Fig. 4a–c, Supplementary Figure 2D). These results suggested that autophagic degradation of Cav-1 emerged, along with reduction of the NO-dependent pathway during the fenestrae shrinking to disappearing, which was consistent with the previous results in the CCl4 rat model. Furthermore, the protein levels of VEGFR2, p-PI3K (Tyr458), PI3K, p-AKT (Ser473), AKT, p-MTOR (Ser2448), and MTOR were decreased, suggested the downregulation of the PI3K–AKT–MTOR pathway (Fig. 4d). Taken together, these data implied that the reduction of the PI3K–AKT–MTOR pathway in LSECs, due to the lack of growth factors in vitro, might trigger autophagic degradation of Cav-1 to promote LSECs defenestration.

### Starvation-induced autophagic degradation of Cav-1 promotes LSECs defenestration

**Fig. 5:**
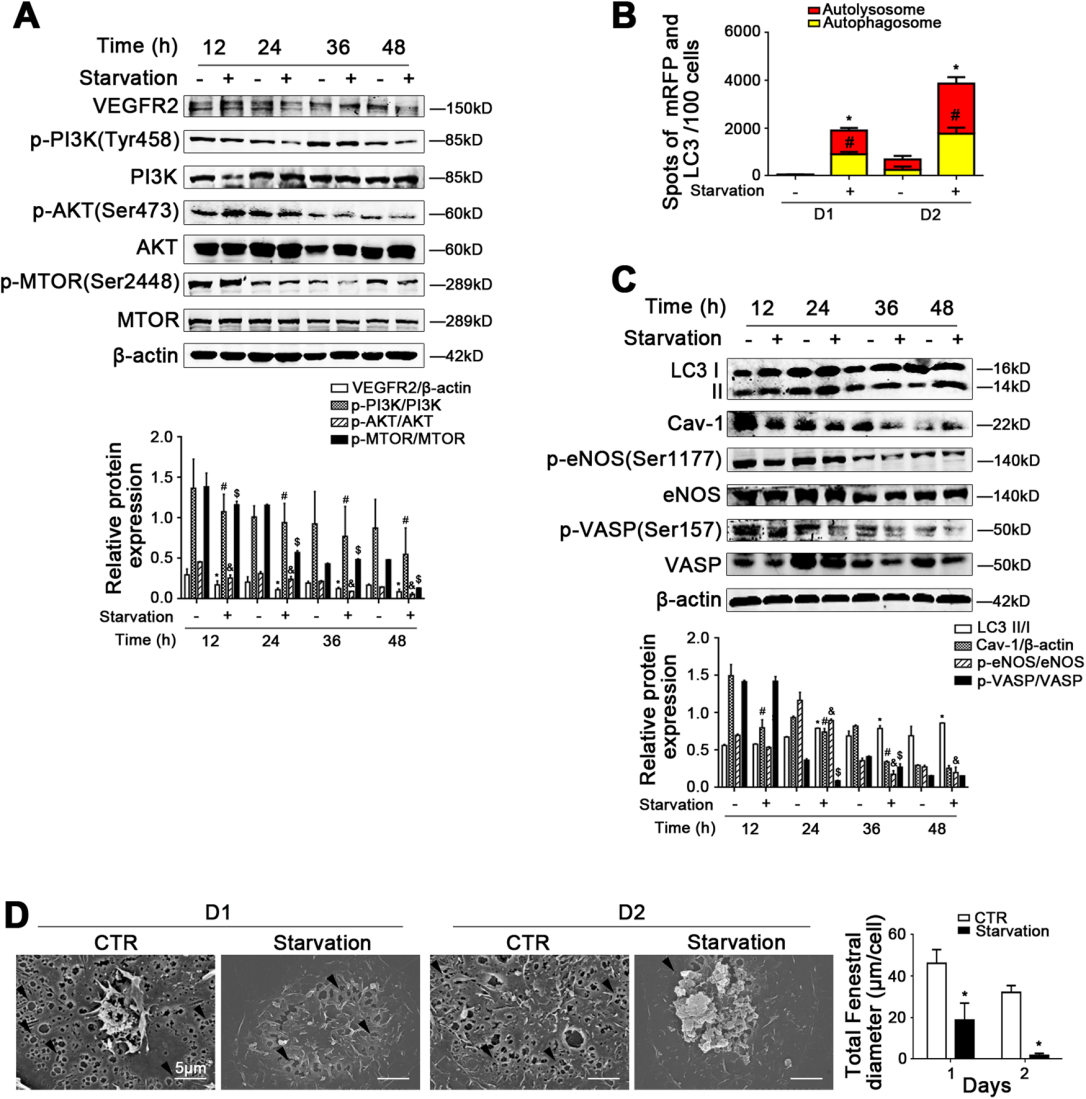
The degradation of Cav-1 is initiated by starvation-induced autophagy to promote LSECs defenestration. Primary rats LSECs, isolated from normal rats, were cultured without fetal bovine serum from 12 h till 48 h in vitro. **a** Protein levels of VEGFR2, p-PI3K (Tyr458), PI3K, p-AKT (Ser473), AKT, p-MTOR (Ser2448), and MTOR analyzed by western blot. The relative protein expression is quantified in the graph, down. *P < 0.05 versus VEGFR2/β-actin of the concurrent control group; ^#^*P* < 0.05 versus p-PI3K/PI3K of the concurrent control group; ^&^*P* < 0.05 versus p-AKT/AKT of the concurrent control group; ^$^*P* < 0.05 versus p-MTOR/MTOR of the concurrent control group. **b** Red or yellow represents autolysosomes or autophagosomes, respectively, visualized by confocal microscopy. Quantification of autophagic flux (%) in 100 cells was analyzed. *P < 0.05 versus the autolysosomes of the concurrent control group; ^#^*P* < 0.05 versus the autophagosomes of the concurrent control group. **c** Protein levels of LC3 II/I, Cav-1, p-eNOS (Ser1177), eNOS, p-VASP (Ser157), and VASP in primary LSECs analyzed by western blot. The relative protein expression is quantified in the graph, down. *P < 0.05 versus LC3 II/I of the concurrent control group; ^#^*P* < 0.05 versus Cav-1/β-actin of the concurrent control group; ^&^*P* < 0.05 versus p-eNOS/eNOS of the concurrent control group; ^$^*P* < 0.05 versus p-VASP/VASP of the concurrent control group. **d** Magnification of SEM of LSECs in the CTR group and the Starvation group for 2 days, revealing the fenestrae structures (scale bar: 5 μm), and quantification of the total fenestral diameter, right. The black triangles indicate LSECs fenestrae structures. *P < 0.05 versus the concurrent control group

Compared to the concurrent control group, the protein expression of VEGFR2, p-PI3K, PI3K, p-AKT, AKT, p-MTOR, and MTOR in LSECs in the starvation group were decreased, along with the augment of autophagic flux and the protein levels of LC3 II/I and Cav-1, suggesting that starvation triggered autophagic degradation of Cav-1 via downregulating the PI3K–AKT–MTOR pathway (Fig. 5a–c). As we had expected, the protein expression of p-eNOS (Ser1177), eNOS, p-VASP (Ser157), and VASP, as well as the data of SEM showed that starvation promoted LSECs defenestration and inhibited the NO-dependent pathway (Fig. 5c, d). It is confirmed that the degradation of Cav-1 is initiated by starvation-induced autophagy, so as to inhibit the NO-dependent pathway and promote LSECs defenestration.

### VEGF maintains LSECs fenestrae via inhibiting autophagic degradation of Cav-1

**Fig. 6:**
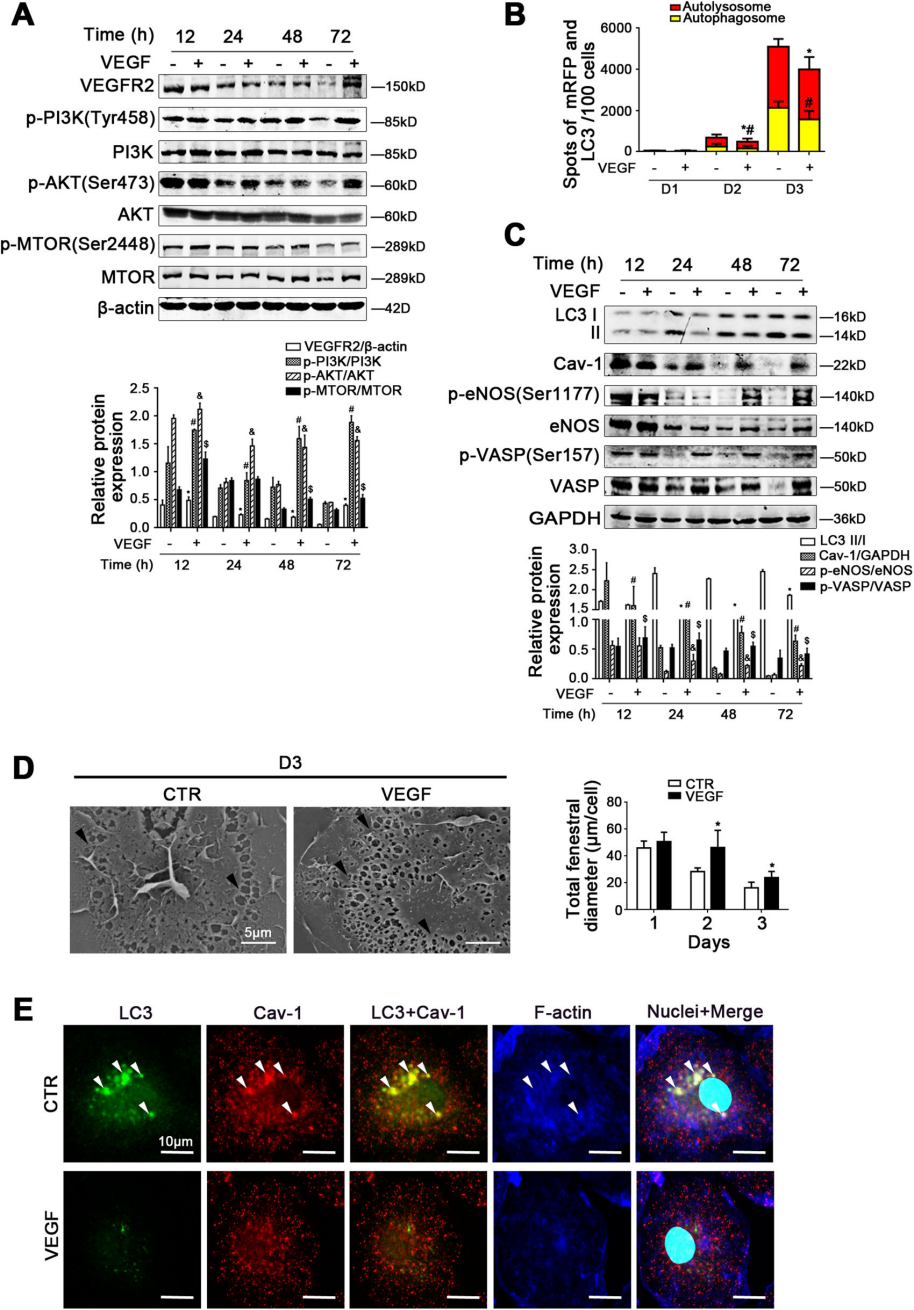
VEGF maintains LSECs fenestrae via inhibiting autophagic degradation of Cav-1. Primary rats LSECs, isolated from normal rats and cultured for 3 days in vitro, were treated with VEGF at the dose of 10 ng/ml from 12 h till 72 h. **a** Protein levels of VEGFR2, p-PI3K (Tyr458), PI3K, p-AKT (Ser473), AKT, p-MTOR (Ser2448), and MTOR analyzed by western blot. The relative protein expression is quantified in the graph, down. *P < 0.05 versus VEGFR2/β-actin of the concurrent control group; ^#^*P* < 0.05 versus p-PI3K/PI3K of the concurrent control group; ^&^*P* < 0.05 versus p-AKT/AKT of the concurrent control group; ^$^*P* < 0.05 versus p-MTOR/MTOR of the concurrent control group. **b** Red or yellow represents autolysosomes or autophagosomes, respectively, visualized by confocal microscopy. Quantification of autophagic flux (%) in 100 cells was analyzed. *P < 0.05 versus the autolysosomes of the concurrent control group; ^#^*P* < 0.05 versus the autophagosomes of the concurrent control group. **c** Protein levels of LC3 II/I, Cav-1, p-eNOS (Ser1177), eNOS, p-VASP (Ser157), and VASP in primary LSECs analyzed by western blot. The relative protein expression is quantified in the graph, down. *P < 0.05 versus LC3 II/I of the concurrent control group; ^#^*P* < 0.05 versus Cav-1/GAPDH of the concurrent control group; ^&^*P* < 0.05 versus p-eNOS/eNOS of the concurrent control group; ^$^*P* < 0.05 versus p-VASP/VASP of the concurrent control group. **d** Magnification of SEM of LSECs in the CTR group and the VEGF group on Day 3, revealing the fenestrae structures (scale bar: 5 μm), and quantification of the total fenestral diameter, right. The black triangles indicate LSECs fenestrae structures. *P < 0.05 versus the concurrent control group. **e** The co-localization of LC3 (green) with Cav-1 (red) and F-actin (blue) in LSECs of the two groups (CTR and VEGF), shown by immunofluorescence. Scale bar: 10 μm

The protein expression of VEGFR2, p-PI3K, PI3K, p-AKT, AKT, p-MTOR, MTOR, LC3 II/I, Cav-1, p-eNOS (Ser1177), eNOS, p-VASP (Ser157), and VASP, as well as the autophagic flux in LSECs, showed that VEGF activated the PI3K–AKT–MTOR pathway to inhibit autophagic degradation of Cav-1 and upregulate the NO-dependent pathway. In contrast, rapamycin pre-treatment could enhance the autophagic degradation of Cav-1 to inhibit the NO-dependent pathway (Fig. 6a–c, Supplementary Figure 3A-C). The data of SEM showed that VEGF maintained LSECs fenestrae, which were deteriorated by rapamycin due to serious autophagy (Fig. 6d, Supplementary Figure 3D). Besides, the immunofluorescence showed that compared with the control group, less LC3 co-localized with Cav-1 and F-actin in the VEGF-treated group; moreover Cav-1 and F-actin were distributed uniformly around the cell membrane, suggesting that the autophagic degradation of Cav-1 and F-actin remodeling were attenuated by VEGF (Fig. 6e). These data confirmed that VEGF could inhibit autophagic degradation of Cav1 and F-actin remodeling, to improve the NO-dependent pathway and maintain LSECs fenestrae.

### Autophagic degradation of Cav-1 promotes LSECs defenestration via inhibiting the NO-dependent pathway

**Fig. 7:**
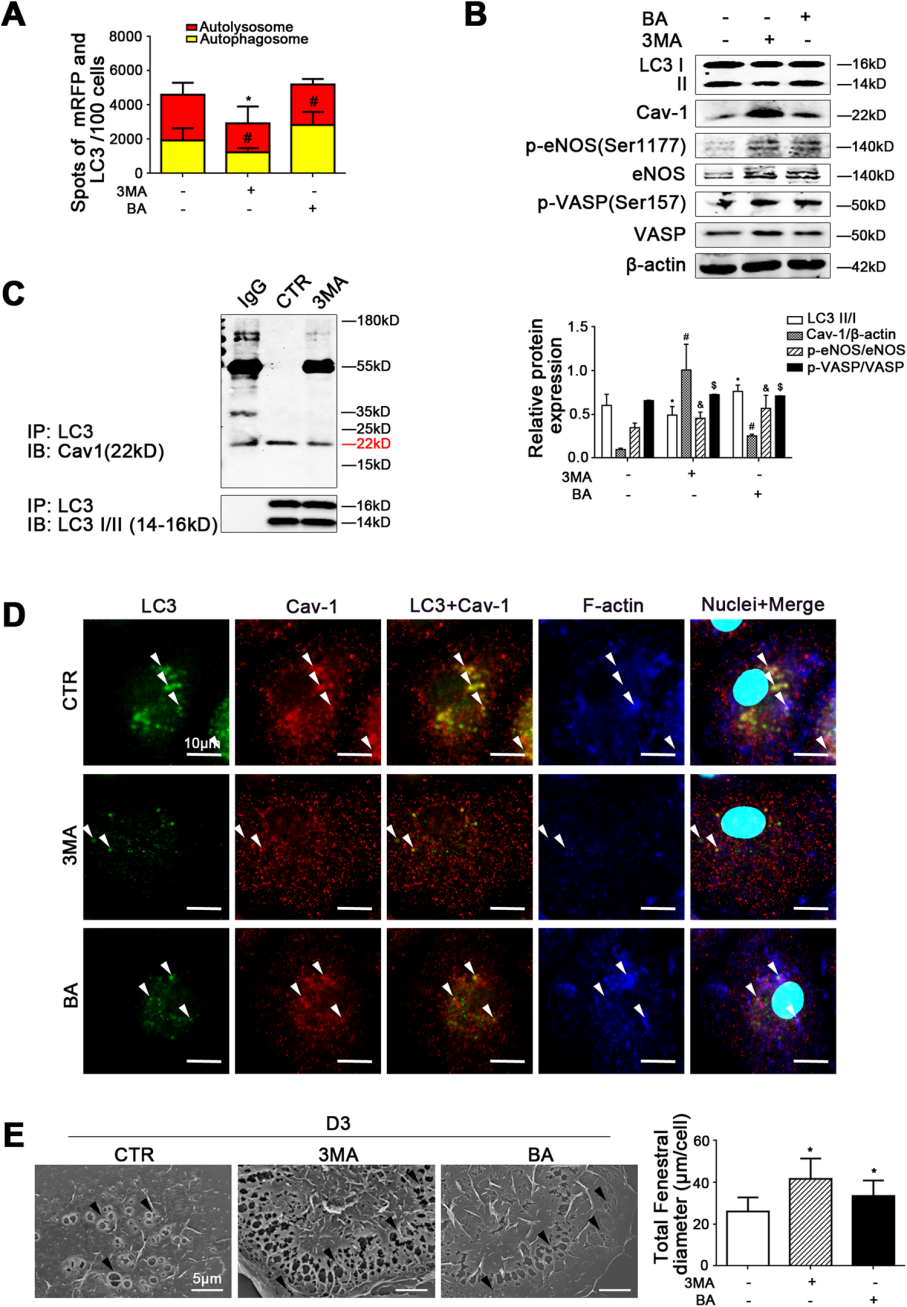
Inhibiting autophagic degradation of Cav-1 could maintain LSECs fenestrae. Primary rats LSECs, isolated from rats and cultured in vitro, were treated with autophagy inhibitors (3MA or bafilomycin) for 3 days. **a** Red or yellow represents autolysosomes or autophagosomes, respectively, visualized by confocal microscopy. Quantification of autophagic flux (%) in 100 cells was analyzed. *P < 0.05 versus the autolysosomes of the control group; ^#^*P* < 0.05 versus the autophagosomes of the control group. **b** Protein levels of LC3 II/I, Cav-1, p-eNOS (Ser1177), eNOS, p-VASP (Ser157), and VASP in primary LSECs analyzed by western blot. The relative protein expression is quantified in the graph, below. *P < 0.05 versus LC3 II/I of the control group; ^#^*P* < 0.05 versus Cav-1/β-actin of the control group; ^&^*P* < 0.05 versus p-eNOS/eNOS of the control group; ^$^*P* < 0.05 versus p-VASP/VASP of the control group. **c** Interaction of LC3 with Cav-1 was detected by the co-IP assay. LC3 of primary LSECs was individually immunoprecipitated and Cav-1 subjected to immunoblotting analysis as indicated. **d** The co-localization of LC3 (green) with Cav-1 (red) and F-actin (blue) in LSECs of the three groups (CTR, 3MA, or bafilomycin), shown by immunofluorescence. Scale bar: 10 μm. **e** Magnification of SEM of LSECs in the three groups (CTR, 3MA, or bafilomycin) on Day 3, revealing the fenestrae structures (scale bar: 5 μm), and quantification of the total fenestral diameter, right. The black triangles indicate LSECs fenestrae structures. *P < 0.05 versus the control group. BA bafilomycin

To further explore the effect of autophagic degradation of Cav-1 on the defenestration, we treated primary LSECs with autophagy inhibitors (3MA or bafilomycin) or knockdown of ATG5. Compared to the control group and the NC group, the autophagic flux, as well as the protein levels of LC3 II/I, Cav-1, p-eNOS, eNOS, p-VASP, and VASP, showed that 3MA, bafilomycin, or siATG5 upregulated the NO-dependent pathway via inhibiting autophagic degradation of Cav-1 (Fig. 7a, b, Supplementary Figure 4A-B). Furthermore, the co-IP assays revealed that less Cav-1 co-precipitated with LC3 in the 3MA-treated group (Fig. 7c), suggesting that 3MA inhibited the interaction of Cav-1 and LC3 due to reduction of autophagy. Similarly, the immunofluorescence showed that in the 3MA- or bafilomycin-treated group, less LC3 co-localized with Cav-1 and F-actin; meanwhile, Cav-1 and F-actin were distributed uniformly around the cell membrane, suggesting that degradation of Cav-1 and F-actin remodeling decreased via inhibition of autophagy (Fig. 7d). Indeed, 3MA, bafilomycin, or siATG5 could maintain LSECs fenestrae (Fig. 7e). These effects suggested that inhibiting autophagic degradation of Cav-1 recovered the NO-dependent pathway and maintained LSECs fenestrae via improving F-actin remodeling.

Interestingly, we discovered that reduction of the NO-dependent pathway by eNOS-siRNA, could promote LSECs defenestration and induce autophagic degradation of Cav-1; in contrast, 3MA could rescue depletion of Cav-1 due to inhibiting autophagy, to maintain LSECs fenestrae (Supplementary Figure 5A-B). Furthermore, the eNOS inhibitor L-NAME promoted LSECs defenestration through downregulation of the NO-dependent pathway, along with aggravating autophagic degradation of Cav-1, which was improved by NO donor (DETA NONOate) (Supplementary Figure 5C-D).

Therefore, autophagic degradation of Cav-1 promoted LSECs defenestration via inhibiting the NO-dependent pathway; in turn, inhibition of the NO-dependent pathway could enhance autophagic degradation of Cav-1 to aggravate disappearance of fenestrae.

### Autophagy regulates LSECs fenestrae mediated by Cav-1

To investigate the role of Cav-1 in autophagy and LSECs phenotype, primary LSECs were transfected with siRNA to knockdown Cav-1. We found that Cav-1-siRNA reduced GLUT3-related glucose uptake and ATP generation, as well as triggered the AMPK-ULK1 pathway and the subsequent increase of the protein levels of ATG5 and LC3 II/I (Supplementary Figure 6A-B). Moreover, Cav-1-siRNA promoted LSECs defenestration via the decline of the NO-dependent pathway (Supplementary Figure 6C-D). Besides, Cav-1-siRNA promoted the co-localization of LC3 and F-actin in the perinuclear area of LSECs (Supplementary Figure 6E). Hence, these effects demonstrated that knockdown of Cav-1 facilitated LSECs defenestration due to the activation of AMPK-dependent autophagy and the subsequent inhibition of the NO-dependent pathway and F-actin remodeling.

**Fig. 8:**
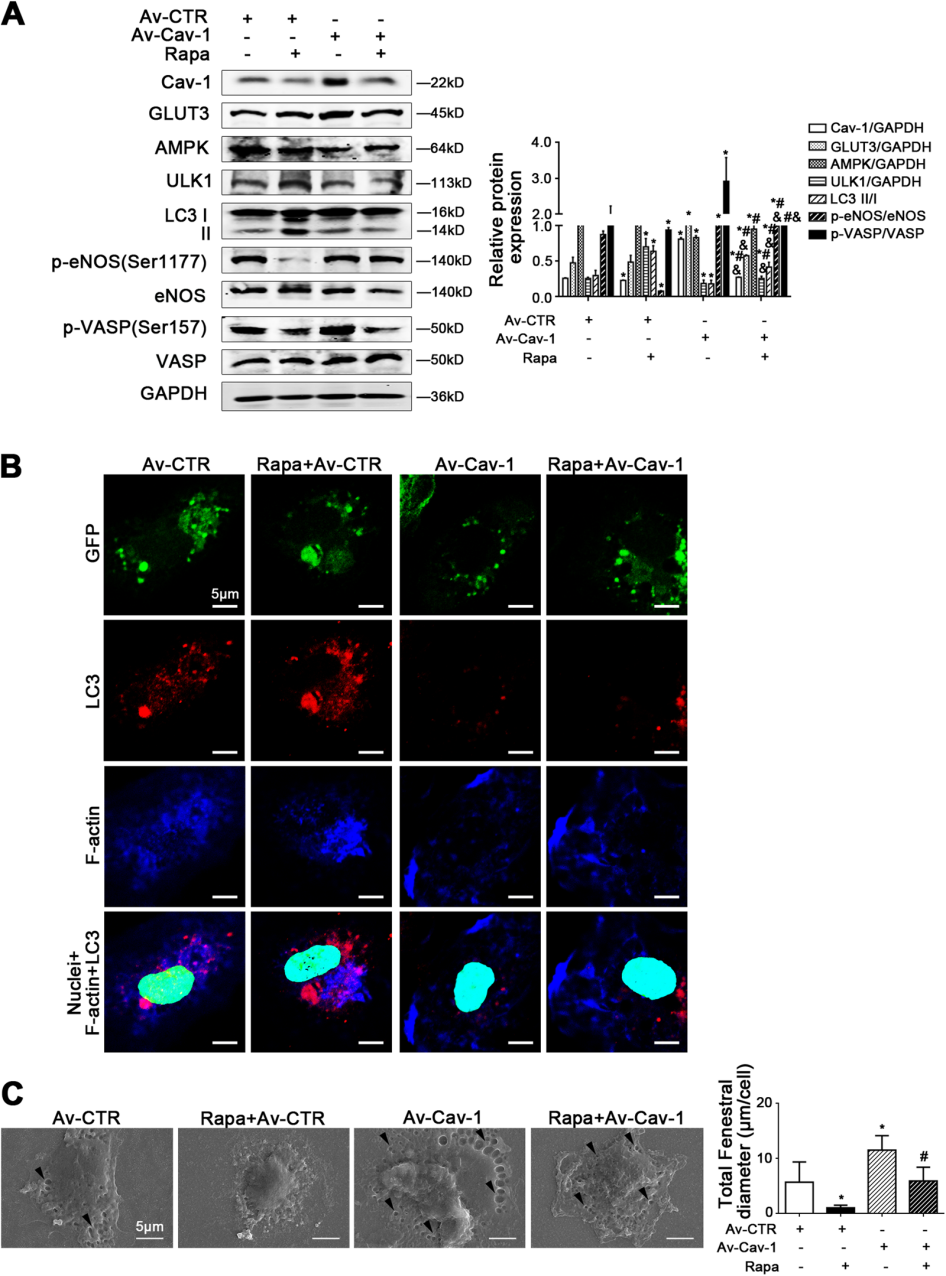
Overexpressed Cav-1 could maintain LSECs fenestrae via rescuing autophagic degradation of Cav-1. Primary LSECs, isolated from rats, were transfected with adenovirus vector to overexpress Cav-1, followed by rapamycin (Rapa)-treatment from days 2 to 4. **a** Protein levels of Cav-1, GLUT3, AMPK, ULK1, LC3 II/I, p-eNOS (Ser1177), eNOS, p-VASP (Ser157), and VASP in primary LSECs analyzed by western blot. The relative protein expression is quantified in the graph, right. *P < 0.05 versus the Av-CTR group; ^#^*P* < 0.05 versus the Av-CTR+Rapa group. **b** The co-localization of LC3 (red) and F-actin (blue) in LSECs of the four groups (Av-CTR, Av-Cav-1, Av-CTR+Rapa, and Av-Cav-1+Rapa), shown by immunofluorescence. Scale bar: 10 μm. **c** Magnification of SEM of LSECs in the four groups (Av-CTR, Av-Cav-1, Av-CTR+Rapa, and Av-Cav-1+Rapa), revealing the fenestrae structures (scale bar: 5 μm), and quantification of the total fenestral diameter, right. The black triangles indicate LSECs fenestrae structures. *P < 0.05 versus the Av-CTR group

Next, primary LSECs were transfected with adenovirus vector to overexpress Cav-1, followed by rapamycin treatment. The data showed that rapamycin treatment aggravated autophagic degradation of Cav-1; in contrast, the overexpressed Cav-1-pre-treatment could rescue the degradation of Cav-1 via enhancing the GLUT3 protein level and inhibit AMPK-ULK1 pathway, to improve the NO-dependent pathway (Fig. 8a). Meanwhile, the immunofluorescence showed that rapamycin enhanced the co-localization of LC3 and F-actin in the perinuclear area of the LSECs, which was inhibited by the overexpressed Cav-1, suggesting that overexpressed Cav-1 could attenuate rapamycin-induced autophagic degradation of Cav-1 and F-actin remodeling (Fig. 8b). Indeed, overexpressed Cav-1 could maintain LSECs fenestrae (Fig. 8c). Hence, knockdown of Cav-1 facilitated defenestration due to the activation of the AMPK-dependent autophagy; whereas, overexpressed Cav-1 rescued rapamycin-induced autophagic degradation of Cav-1 to maintain LSECs fenestrae.

### Rapamycin-induced defenestrated LSECs activated HSCs due to enhanced autophagy

Primary rats LSECs were pre-treated by rapamycin for 3 days, followed by co-culture with fresh primary rats HSCs for 3 days and 6 days. Quiescent HSCs, co-cultured with rapamycin-induced defenestrated LSECs for 3 days and 6 days, were more activated, indicating activation of HSCs by defenestrated LSECs due to serious autophagy (Supplementary Figure 7).

## Discussion

**Fig. 9:**
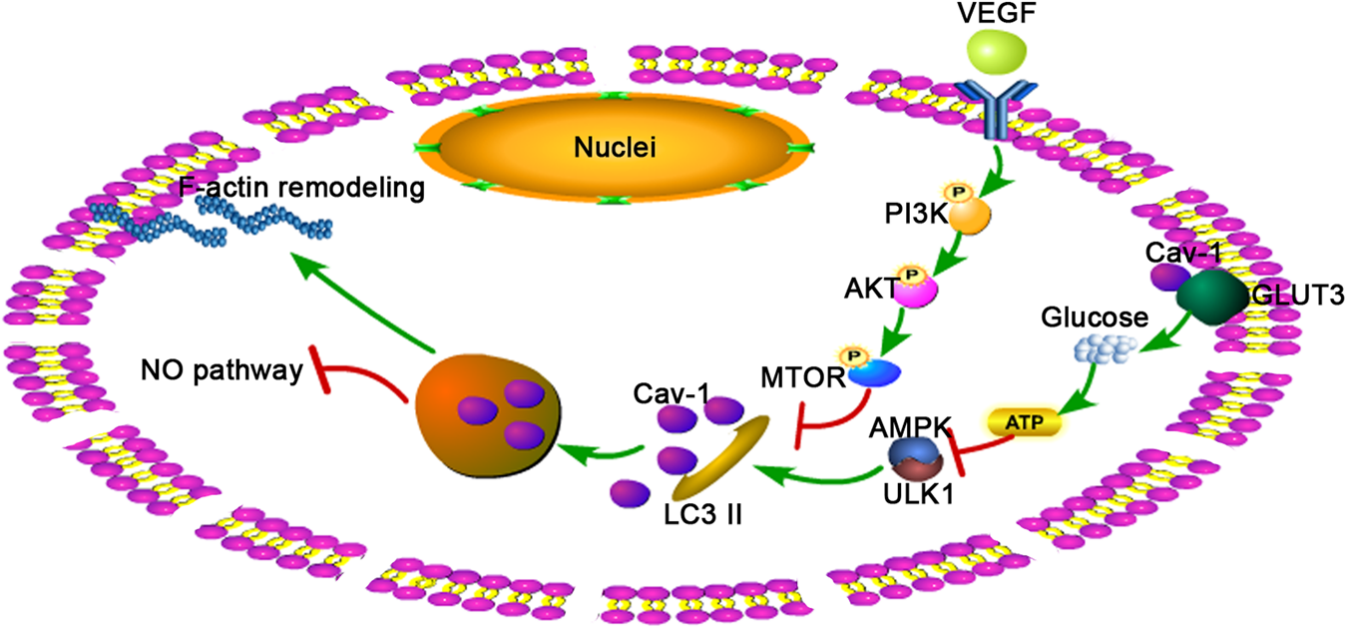
**A schematic view of major signal transduction pathways involved in the conclusion that autophagic degradation of Cav-1 promotes LSECs defenestration via F-actin remodeling and inhibition of the NO-dependent pathway**

In the present study, we demonstrated that autophagic degradation of Cav-1 promoted F-actin remodeling and inhibited the NO-dependent pathway to aggravate LSECs defenestration via downregulation of the PI3K–AKT–MTOR pathway (Fig 9). The primary findings include the following: 1. Autophagy initiated the degradation of Cav-1 during CCl4-induced LSECs defenestration and liver fibrogenesis; in contrast, autophagic inhibitor 3MA could inhibit autophagic degradation of Cav-1 to maintain fenestrae and attenuate liver fibrosis. 2. Autophagic degradation of Cav-1 promoted LSECs defenestration through inhibition of the PI3K–AKT–MTOR pathway, which was aggravated by starvation. 3. VEGF maintained LSECs fenestrae and improved the NO-dependent pathway via activation of the PI3K–AKT–MTOR pathway and subsequent inhibition of autophagic degradation of Cav-1 and F-actin remodeling. 4. Overexpressed Cav-1 rescued the depletion of Cav-1 to maintain LSECs fenestration via inhibiting autophagy and F-actin remodeling.

Differentiated LSECs (namely fenestrated LSECs) function, as a gatekeeper, could prevent the activation of HSCs; however, once LSECs defenestrate and capillarize, it perhaps promotes the activation of HSCs to enhance the progression of fibrosis^3^. Therefore, it is important to investigate the underlying mechanism and the intervening target of LSECs defenestration in liver fibrogenesis. In the progression and aggravation of liver diseases, autophagy plays different roles in phenotype and function of intra-hepatic cells. HSCs, as a key for liver fibrosis, could be activated by serious autophagy to aggravate fibrogenesis^11^. Interestingly, some literatures regarding the influence of autophagy on LSECs phenotype and function are controversial. It is reported that statins improve hepatic endothelial function and paracrine endothelial-stellate cell activation in CCl4-cirrhotic rats mediated by KLF2, involved in endothelial vasoprotection^22,23^. Besides, in acute liver injury induced by ischemia/reperfusion (I/R), statins could activate autophagy to protect LSECs microvascular function mediated by KLF2, indicating the beneficial effects of autophagy on acute liver injury^14^.

However, our previous studies reveal that in the early stage of liver fibrosis, autophagy, initiated by aldosterone-induced oxidation, promotes LSECs defenestration^15^. Here, we further found that serious autophagy emerged in LSECs in human fibrotic liver. During the initiation of fibrosis in vivo, CCl4 induced defenestration, a pro-fibrotic phenotype transition of LSECs, along with a time-dependent elevation of autophagy and depletion of Cav-1 in LSECs; while autophagy inhibitor 3MA inhibited these effects to maintain fenestrae and attenuated liver fibrosis. Moreover, in vitro, the degradation of Cav-1 induced by autophagy promoted LSECs defenestration. Starvation, confirmed as a golden standard for initiating autophagy^24^, accelerates LSECs defenestration due to augment of autophagic degradation of Cav-1. However, autophagy inhibitors (3MA or bafilomycin) and knockdown of ATG5 maintain LSECs fenestrae due to reduction of autophagic degradation of Cav-1. In addition, autophagy activator rapamycin induced LSECs defenestration; furthermore, these defenestrated and dysfunctional LSECs, induced by serious autophagy, could activate HSCs to initiate liver fibrogenesis. Hence, autophagic degradation of Cav-1 is a promising intervention target for LSECs defenestration and the early stage of fibrosis.

Next, we further explore the mechanisms of autophagic degradation of Cav-1 in LSECs defenestration. Petra Krause and colleagues^25^ find that the numerous sieve plates of isolated LSECs gradually disappeared during culture without growth factors in vitro^26^. Moreover, primary LSECs had no visible fenestrae in serum-free cultural condition^21^. Indeed, we also demonstrated that without growth factors or in a serum-free condition, LSECs fenestrae shrank to disappear rapidly, along with the downregulation of the PI3K–AKT–MTOR pathway. As we know that VEGF, confirmed for maintaining LSECs fenestrae^26,27^, could inhibit autophagy via activating the VEGFR2–PI3K–AKT–MTOR signaling pathway^28,29^. Our data showed that VEGF inhibited autophagic degradation of Cav-1 to upregulate the NO-dependent pathway, improve F-actin remodeling, and maintain LSECs fenestration via activation of the PI3K–AKT–MTOR pathway; while rapamycin co-treatment increased degradation of Cav-1 to promote defenestration. Therefore, VEGF reduces autophagic degradation of Cav-1 via upregulation of the PI3K–AKT–MTOR pathway, to maintain fenestrae.

Additionally, it is confirmed that the NO-dependent (namely the NO/eNOS/sGC/cGMP/PKG/VASP) pathway is the classic pathway required for maintaining LSECs fenestrae^20^. Meanwhile, the NO-independent pathway is growing into an attractive regulating mechanism and is connected to autophagy. It is reported that eNOS inhibitor could enhance autophagosome synthesis and autophagosome–lysosome fusion^30^. Rapamycin, an autophagy enhancer, could negatively regulate the protein expression of eNOS and NO synthesis in endothelial cells^31^. Our results for the first time demonstrated the interaction of autophagy with the NO-dependent pathway for LSECs phenotype. We found that autophagy inhibitors (3MA or bafilomycin) attenuated autophagic degradation of Cav-1 and improved the NO-dependent pathway to maintain LSECs fenestrae. In contrast, eNOS-siRNA and eNOS inhibitor L-NAME, for inhibiting the NO-dependent pathway, could induce autophagic degradation of Cav-1 and promote LSECs defenestration. Therefore, serious autophagic degradation of Cav-1 negatively regulates the NO-dependent pathway to promote LSECs defenestration; in turn, inhibition of the NO-dependent pathway could enhance autophagic degradation of Cav-1 to aggravate disappearance of fenestrae.

Finally, we focus on the role of distribution of Cav-1, induced by autophagy, in regulating LSECs fenestration. Cav-1, an important structure protein around the fenestrae and on vesicles, participates in signal transduction. However, literatures about the effect of Cav-1 on fenestrae are diverse. It is reported that Cav-1 is closely affiliated with LSECs fenestration^4,6^. Nevertheless, Warren et al. demonstrated that LSECs fenestrae of Cav-1-knockout mice were not changed under the normal conditions^32^. However, Cav-1 is a key molecule for regulating LSECs fenestration via energetic balance, and autophagy. Yokomori et al.^26^ also noted that during LSECs phenotype maintaining, Cav-1-positive aggregates in the cytoplasm and reactivity on vesicles and vacuoles prominently increased, especially surrounding the nuclear region in LSECs. Nevertheless, our previous studies confirmed that the Cav-1 protein level in the membrane and the cytoplasm simultaneously declined in rapamycin-treatment group to facilitate LSECs defenestration, due to serious autophagy^15^. Furthermore, during the process of LSECs defenestration, serious autophagy, induced by starvation (namely serum-free treatment) and rapamycin treatment, triggered the assemblage of Cav-1 to the nuclear region and degradation of Cav-1. VEGF treatment, which maintained LSECs fenestrae, could attenuate autophagic degradation and redistribution of Cav-1. Cav-1 distributes uniformly around the cell membrane in VEGF-, 3MA-, or bafilomycin-treatment due to the reduction of autophagy. However, the function of Cav-1 redistribution in LSECs phenotype needs further elucidation.

In addition, Cav-1 also mediates autophagy through regulation of energetic generation. Ha et al.^17^ determined that depletion of Cav1 led to reduction of GLUT3-related glucose uptake and ATP generation, activating AMPK-ULK1 pathway to induce autophagy and diminish cellular metabolism, which in turn reinforced AMPK-dependent autophagy. Moreover, Cav-1 connects with the ATG12-ATG5 system to suppress autophagy^19^. We found that knockdown of Cav-1 led to defenestration due to initiation of autophagy mediated by the reduction of GLUT3 and ATP. Notably, Cav-1-siRNA promoted LC3 co-localization with F-actin in the perinuclear area of LSECs, suggesting a link between autophagy and F-actin remodeling. As mentioned above, it is worthy to note that serious autophagy induces the degradation of Cav-1; in turn, the depletion of Cav-1 also reinforces autophagy, perhaps through at least two ways: (1) The AMPK-dependent autophagy induced by the decrease of GLUT3 and low ATP. (2) The increase of ATG5 initiated by autophagic degradation of Cav-1. Furthermore, rapamycin treatment aggravated autophagic degradation of Cav-1; in contrast, the overexpressed Cav-1 treatment could rescue the depletion of Cav-1 via inhibiting AMPK-ULK1 pathway, to attenuate LSECs defenestration.

There are still some limitations to our present research. Whether the function of Cav-1 varies with the location of Cav-1 is still unknown. Furthermore, the mechanism about the degradation and redistribution of Cav-1 in the regulation of F-actin remodeling and LSECs defenestration needs to be explored deeply.

Taken together, we identified Cav-1 as a crucial factor that is associated with energetic balance, autophagy, F-actin remodeling, and the NO-dependent pathway. Autophagic degradation of Cav-1, due to the downregulation of the PI3K–AKT–MTOR pathway, promoted LSECs defenestration by inhibiting the NO-dependent pathway and initiating F-actin remodeling. The present study revealed that inhibiting autophagic degradation of Cav-1 is a promising strategy for preventive treatment of defenestration.

## Materials and methods

### Reagents and antibodies

The reagents used included 3MA (Sigma-Aldrich, S2767), rapamycin (Sigma-Aldrich, S1039), bafilomycin A1 (Sigma-Aldrich, SML1661), VEGF (PeproTech, 100-20A), N-Nitro-L-Arginine Methyl Ester (L-NAME, Sigma-Aldrich, N5751), Diethylenetriamine/nitric oxide adduct (2,2′-(Hydroxynitrosohydrazono)bis-ethanimine DETA/NO, NO donor, Sigma-Aldrich, D185).

The antibodies used included anti-Col I (Proteintech, 14695-1-AP), anti-α-SMA (Boster, BM0002), anti-vWF (Santa Cruz, SC-365712), anti-CD31 (Santa Cruz, SC-46694), anti-Cav-1 (Abcam, ab17052), anti-Cav-1 (Abcam, ab2910), anti-LC3 (Abcam, ab48394), anti-p-VASP (Ser157) (CST, 84519), anti-VASP (CST, 3132S), anti-p-eNOS (Ser1177) (CST, 9570), anti-eNOS (Abclonal, A1548), anti-p-PI3K (Tyr458) (CST, 4228), anti-PI3K (CST, 4249), anti-p-AKT (Ser473) (CST, 4060), anti-AKT (CST, 2938), anti-p-MTOR (Ser2448) (CST, 5536), anti-MTOR (CST, 2983), anti-GLUT3 (Abcam, ab41525), anti-AMPK (Proteintech, 10929-2-AP), anti-ULK1 (Proteintech, 20986-1-AP), anti-GAPDH (Proteintech, 60004-1), and anti-β-actin (Proteintech, 60008-1). DAPI (Sigma-Aldrich, D9542), FITC-labeled goat anti-rabbit IgG (H+L) (Beyotime, a0562), Cy3-labeled goat anti-mouse IgG (H+L) (Beyotime, a0521), and phallotoxins (Thermo, F432) were also used.

### Patients

Fibrotic liver biopsy specimens (fibrosis stage: F3–4) were obtained from 13 patients with liver fibrosis due to HBV infection. Normal liver specimens were obtained from nine patients who underwent a partial liver resection for hepatic hemangioma. All patients signed the informed written consent, and the Ethics Committee at the local hospital approved the use of samples.

### Animal experimental design

Sprague–Dawley (SD) rats were provided by the Laboratory Animal Center (Southern Medical University, China) and were approved by the Committee on the Ethics of Animal Experiments of Southern Medical University. Animals were housed under a 12:12 h light/dark cycle at 22–24 °C.

#### CCl4-induced liver fibrosis rat model

Male SD rats (180–200 g) were subjected to intraperitoneal injection of a 40% carbon tetrachloride (CCl4)–olive oil solution at 2 ml/kg body weight, twice a week for 28 days. At Days 0, 3, 6, 14, and 28, CCl4-induced rats were randomly sacrificed (*n* = 4 per group). Besides, to investigate the role of autophagy in liver fibrosis, we employed the CCl4-induced liver fibrosis rat models (*n* = 6 for 6 days and *n* = 6 for 28 days). The vehicle group (*n* = 6 for 6 days and *n* = 6 for 28 days) was subjected to intraperitoneal injection of the same volume of olive oil, twice a week for 28 days. The 3MA-treated group (*n* = 6 for 6 days and *n* = 6 for 28 days) was subjected to intraperitoneal injection of CCl4–olive oil solution twice a week and 3MA (10 mg/kg) per day. The CCl4-induced rat models and the vehicle group were subjected to intraperitoneal injection of normal saline in the same volume every day.

### Histological analysis and immunohistochemistry

Paraffin sections (4 μm) of human or rat liver tissues were prepared with hematoxylin and eosin (H&E) staining and Sirius Red staining. Immunohistochemical detection of Col I, CD31, α-SMA, and vWF was performed on paraffin sections (4 μm), and subsequent sections were exposed to HRP-antibody colored with DAB, and visualized by microscopy (BX51, Olympus, Japan). The degree of liver fibrosis and the number of positive cells were quantified with Image J software.

### Fluorescence staining

Paraffin sections (4 μm) were prepared for immunofluorescence, incubated with primary antibody overnight, followed by the secondary antibody, and then mounted with DAPI. The primary antibodies included anti-vWF (1:100), anti-Cav-1 (1:200), and anti-LC3 (1:200). The secondary antibodies included FITC-labeled goat anti-rabbit IgG (H+L) (1:200) and Cy3-labeled goat anti-mouse IgG (H+L) (1:200).

### SEM and TEM

The primary LSECs and liver tissues were fixed with 2.5% glutaldehyde and subsequently dehydrated and then coated with gold using the coating apparatus. Eventually, the LSECs fenestrae of samples were observed with SEM at 15-kV acceleration voltage. Additionally, the samples for TEM were stained with uranyl acetate and lead citrate, and autophagosomes and autolysosomes were observed using TEM at an 80-kV acceleration voltage.

### Primary LSECs isolation, culture, and treatment

Primary LSECs and HSCs were isolated from male SD rats, based on a modified method^15,33^. LSECs were cultured in plates with medium comprising 40% MCDB131 (Gibco, 10372019), 40% 1640 (Gibco, 11875101), and 20% fetal bovine serum (FBS, TransSerum, 10102). Primary LSECs were treated with VEGF (10 ng/ml), rapamycin (10 nM), 3MA (10 μM), bafilomycin A1 (10 nM), L-NAME (3 mM), and NO donor (DETA NONOate, 6 μM) for 3 days.

### Monitoring LSECs autophagic flux

Primary rat LSECs were transfected with an mRFP-GFP-LC3 encoding plasmid (Hanbio, LP2100001). Transfection efficiency was 70%. To detect autophagy flux in LSECs, the above treatments were used, and autophagic flux was visualized with confocal microscopy (Fluoview FV10i, Olympus, Japan).

### Immunocytochemistry

Paraformaldehyde-fixed primary cells were incubated with primary antibody, followed by the secondary antibody, and subsequently mounted with DAPI. The primary antibodies included anti-Cav-1 (1:50) and anti-LC3 (1:200). To detect F-actin, after incubation with primary antibody and the secondary antibody, the cells were stained with phallotoxins (Thermo, F432). The number of puncta per cell or positive cells was observed by fluorescence microscopy (1X71, Olympus, Japan) and quantified by Image J software.

### Adenoviral vector and transfection

The recombinant adenovirus was produced by Genchem AdenoVector Institute (Shanghai, China). To construct green fluorescence protein-tagged Cav-1, full-length Cav-1 cDNA was amplified from a human cDNA library and fused at its C-terminus with sequences encoding monomeric green fluorescence protein. Briefly, the amplified Cav-1 fragment was inserted into the adenoviral vector, 3 pBHG lox ΔE1, 3 Cre, which contains the mouse cytomegalovirus (CMV) promoter, using the AdMax system. The resultant Cav-1-green fluorescence protein gene fusion was validated by nucleotide sequencing. Transfection efficiency, which was assessed by fluorescence imaging and immunoblotting (IB), was 70–80%, respective of the amount of plasmid used in the transfection. A nonspecific plasmid encoding β-galactosidase was used to maintain identical amounts of DNA in each transfection. Primary LSECs were transfected with this adenovirus vector to overexpress Cav-1, according to the manufacturer's instructions.

### Small interfering RNA (siRNA) transfection assay

Primary LSECs were transfected with siRNA to knockdown Cav-1, ATG5, and eNOS according to the manufacturer's instructions. The transfection efficiency was 70%. The following Cav-1 siRNA sequences were used: sense (5′-GUUGUACCGUGCAUCAAGATT-3′), antisense (5′-UCUUGAUGCACGGUACAACTT-3′). The following ATG5 siRNA sequences were used: sense (5′-CAUGUGUGAAGGAAGCUGATT-3′), antisense (5′-UCAGCUUCCUUCACACAUGTT-3′). The following eNOS siRNA sequences were used: sense (5′-GGUUAUACGACGAUCUUUATT-3′), antisense (5′-UAAAGAUCGUCGUAUAACCTT-3′).

### q-RT-PCR

Total RNA of cells (10^6^) was extracted with Trizol and was reverse transcribed with PrimeScriptTM RT Maseter Mix (Takara, RR036A). Real-time PCR was performed with SYBR Premix Ex TaqTM IIz (Takara, RR820A), using a ROCHE LightCycler® 480. The samples were analyzed using the 2^−△△Ct^ method from the Ct values of the respective RNAs (sGC, cGMP, and PKG) relative to the housekeeping gene GAPDH. The following primers were used. sGC primer (sense: 5′- ACGGAGCCAATAGAGGAGGT-3′; antisense: 5′- TGCCAGGGTGTGTAGGTAGA-3′); cGMP primer (sense: 5′-ATTGGGATTGTGGGCCATGT-3′; antisense: 5′-TTGTCACGTAGTCGGTGAGC-3′); PKG primer (sense: 5′-CGTTACCCGAGAAGACTCACC-3′; antisense: 5′-GAGACATCATCCAGTCCTCCAA-3′).

### Western blotting

The protein expression in liver tissue or primary LSECs was detected by western blot. The primary antibodies included anti-Col I (1:1000), anti-α-SMA (1:1000), anti-CD31 (1:500), anti-Cav-1 (1:1000), anti-LC3 (1:1000), anti-p-VASP (1:1000), anti-VASP (1:1000), anti-p-eNOS (1:1000), anti-eNOS (1:1000), anti-p-PI3K (1:1000), anti-PI3K (1:1000), anti-p-AKT (1:1000), anti-AKT (1:1000), anti-p-MTOR (1:1000), anti-MTOR (1:1000), anti-AMPK (1:1000), anti-ULK1 (1:1000), anti-GLUT3 (1:1000), anti-GAPDH (1:1000), and anti-β-actin (1:1000). The secondary antibodies were donkey anti-mouse (1:15000, LI-COR biosciences, C40910-04) and goat anti-rabbit (1:15000, LI-COR biosciences, C51007-08).

### Co-immunoprecipitation

Primary LSECs were treated with 3MA for 3 days. Immunoprecipitation (IP) and IB were performed as previously described^34^. The antibodies for IP included anti-LC3; the antibodies for IB included anti-Cav-1 and anti-LC3.

### The ATP assay

An ATP Assay Kit (Beyotime, S0026) was used to measure the ATP level in the cells, according to the manufacturer’s protocol.

### Co-culture of LSECs and HSCs

Primary rat LSECs, treated by rapamycin for 3 days, co-cultured with fresh primary HSCs isolated from male SD rats for 3 and 6 days. Quiescent HSCs were labeled by anti-desmin (1:100). Activated HSCs were labeled by anti-α-SMA (1:150). The number of desmin or α-SMA-positive cells was visualized by confocal microscopy.

### Statistics

The experimental data are reported as the mean ± SD. In statistical analysis of two groups, a two-tailed Student’s *t* test was utilized. In the analysis of more than two groups, ANOVA analyses were performed and analyzed by SPSS18.0 software and *P* < 0.05 was considered significant.

## Electronic supplementary material

Supplementary

Supplementary figure 1

Supplementary figure 2

Supplementary figure 3

Supplementary figure 4

Supplementary figure 5

Supplementary figure 6

Supplementary figure 7
