# Author Correction: Autophagic degradation of caveolin-1 promotes liver sinusoidal endothelial cells defenestration

**DOI:** 10.1038/s41419-019-1722-y

**Published:** 2019-06-17

**Authors:** Xiaoying Luo, Dan Wang, Xintao Zhu, Guozhen Wang, Yuehua You, Zuowei Ning, Yang Li, Siyi Jin, Yun Huang, Ye Hu, Tingting Chen, Ying Meng, Xu Li

**Affiliations:** 1 State Key Laboratory of Organ Failure Research, Guangdong Provincial Key Laboratory of Viral Hepatitis Research, Department of Infectious Diseases, Nanfang Hospital, Southern Medical University, Guangzhou, China; 2 Guangdong Provincial Key Laboratory of Gastroenterology, Department of Gastroenterology, Nanfang Hospital, Southern Medical University, Guangzhou, China; 3 Department of Stomatology, People’s hospital of Longhua, Shenzhen, Guangdong China; 4 Department of Respiratory Diseases, Nanfang Hospital, Southern Medical University, Guangzhou, China

**Correction to: Cell Death and Disease**

10.1038/s41419-018-0567-0 published online 14 May 2019

After publication of this article, it was realized that for the authors Xiaoying Luo, Dan Wang, Xintao Zhu and Xu Li the Nanfang Hospital at Southern Medical University was accidentally omitted from their affiliation designation. The correct full affiliation for the State Key Laboratory of Organ Failure Research is:

State Key Laboratory of Organ Failure Research, Guangdong Provincial Key Laboratory of Viral Hepatitis Research, Department of Infectious Diseases, Nanfang Hospital, Southern Medical University, Guangzhou, China.

The authors apologize for this omission.

