# Supplementary material for: Autophagic degradation of caveolin-1 promotes liver sinusoidal endothelial cells defenestration
Source: Cell Death Dis. 2018 May 14;9(5):576. doi: 10.1038/s41419-018-0567-0 (PMC5951836; doi:10.1038/s41419-018-0567-0)
Supplement: Supplementary file 1 — Supplementary [file 41419_2018_567_MOESM1_ESM.doc]

**Supplementary**

**Supplementary figure legends:**

**
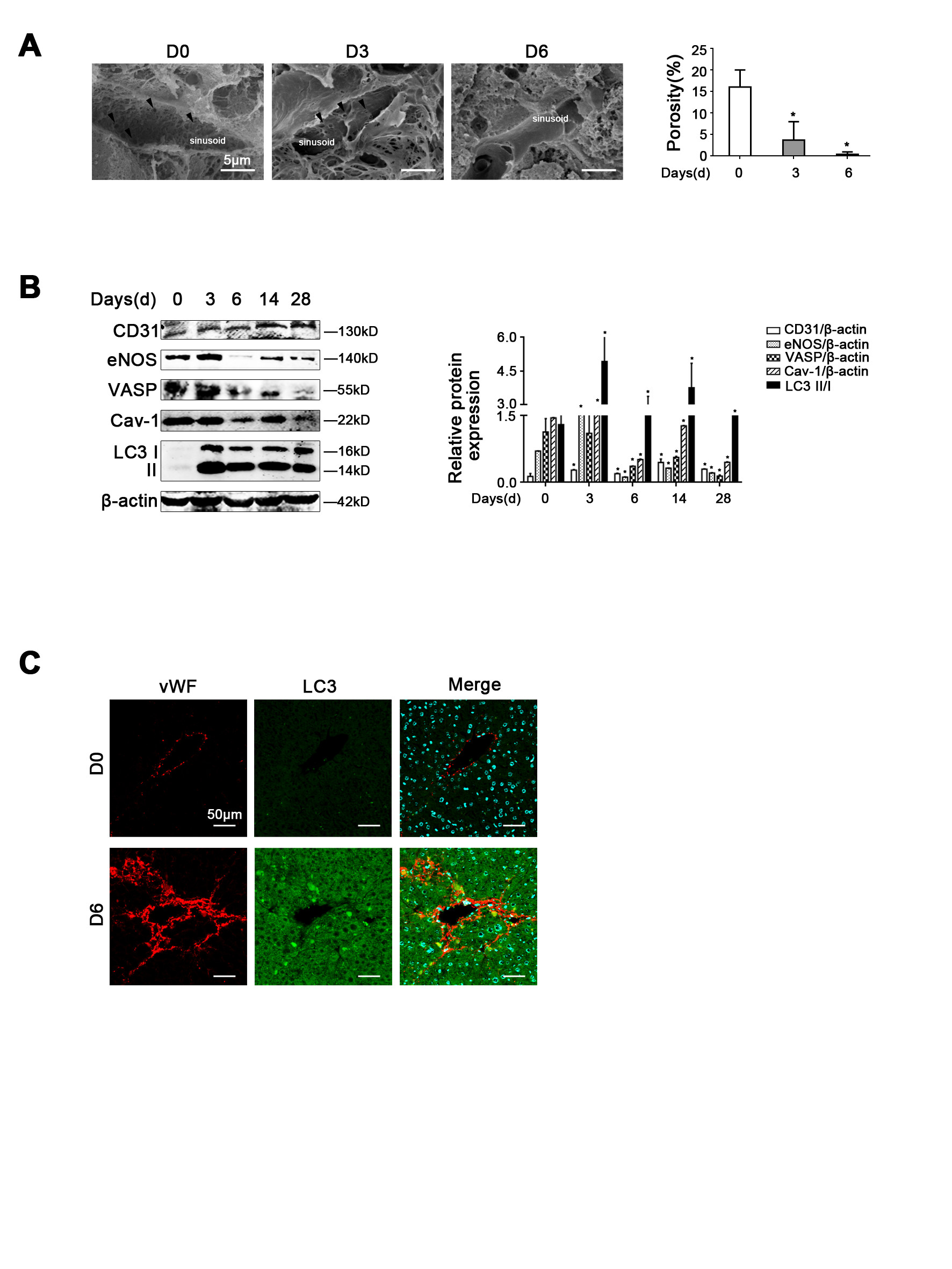
**

**Supplementary Figure 1. Cav-1 was degraded by serious autophagy in the process of CCl4-induced LSECs defenestration.** At Days 0, 3, 6, 14, and 28, CCl4-induced rats were randomly sacrificed (n=4 per group). (**A**) Magnification of SEM of rat liver sinusoidal endothelium at different time points (Day 0, Day 3, and Day 6) in the progression of CCl4-induced liver fibrosis (Scale bar: 5 μm), and quantification of porosity in sinusoidal endothelium, right. The black arrows indicate LSECs fenestrae structures. *P<0.05 versus Day 0. (**B**) Representative immunoblots of CD31, eNOS, VASP, Cav-1, and LC3 II/I of primary LSECs isolated from the CCl4 rat model at Days 0, 3, 6, 14, and 28. The relative protein expression is quantified in the graph, right. *P<0.05 versus Day 0. (**C**) The co-localization of LC3 with vWF by immunofluorescence (Scale bar: 50 μm).


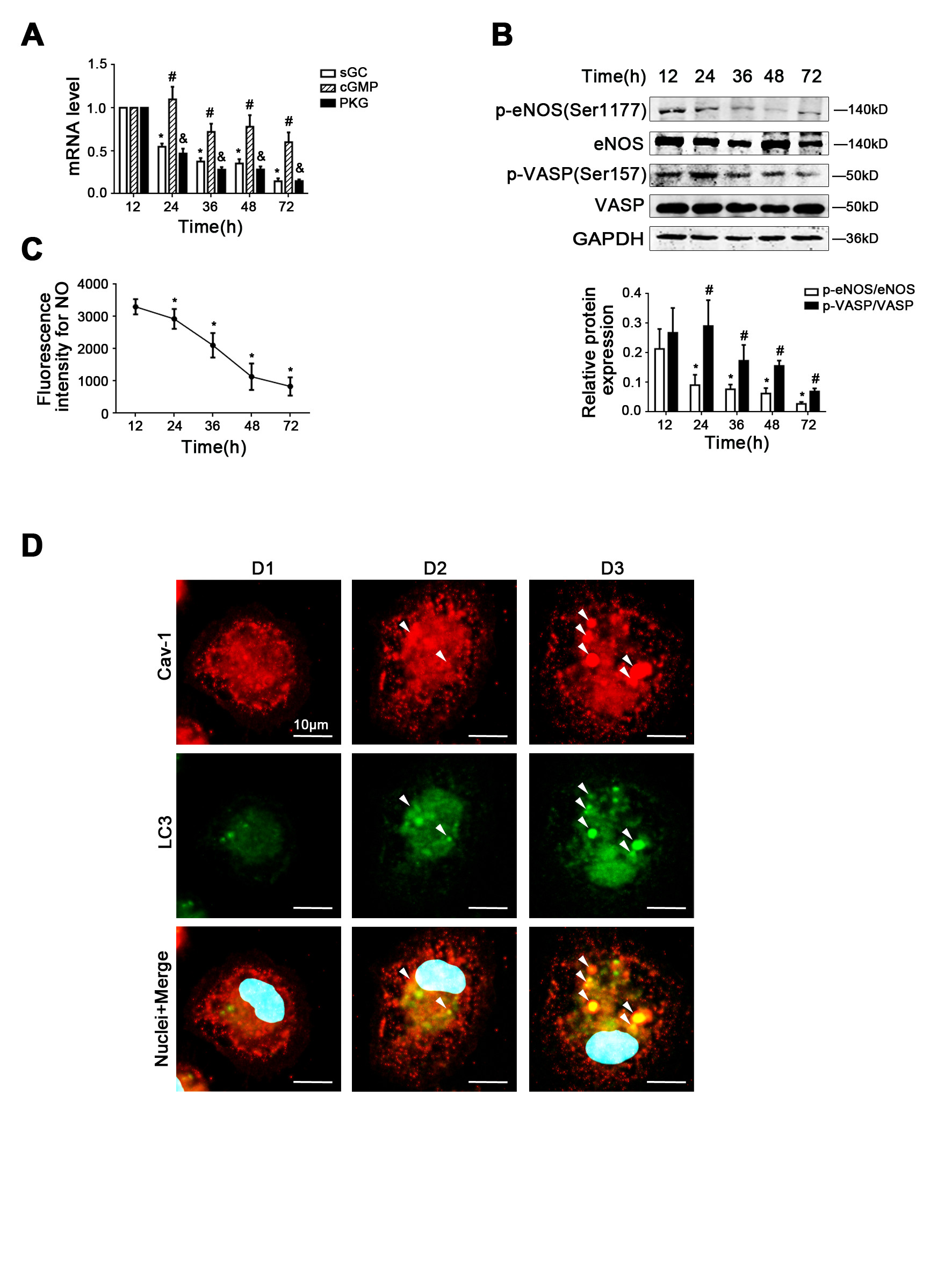


**Supplementary Figure 2. Autophagic degradation of Cav-1 was triggered, along with reduction of the NO-dependent pathway during the progression of LSECs defenestration.** Primary LSECs, isolated from normal rats, were cultured for 3 days *in vitro*. (**A**) Real-time PCR analysis of sGC, cGMP, and PKG mRNA levels in LSECs. *P<0.05 versus sGC mRNA level of the 12h group; #P<0.05 versus cGMP mRNA level of the 12h group; &P<0.05 versus PKG mRNA level of the 12h group. (**B**) Protein levels of p-eNOS (Ser1177), eNOS, p-VASP (Ser157), and VASP in primary LSECs analyzed by western blot. The relative protein expression is quantified in the graph, down. *P<0.05 versus p-eNOS/eNOS of the 12h group; #P<0.05 versus p-VASP/VASP of the 12h group. (**C**) The quantification of NO level in LSECs. *P<0.05 versus the 12h group. (**D**) The co-localization of LC3 (green) with Cav-1 (red) in LSECs from Day 1 till Day 3, shown by immunofluorescence. Scale bar: 10 μm.


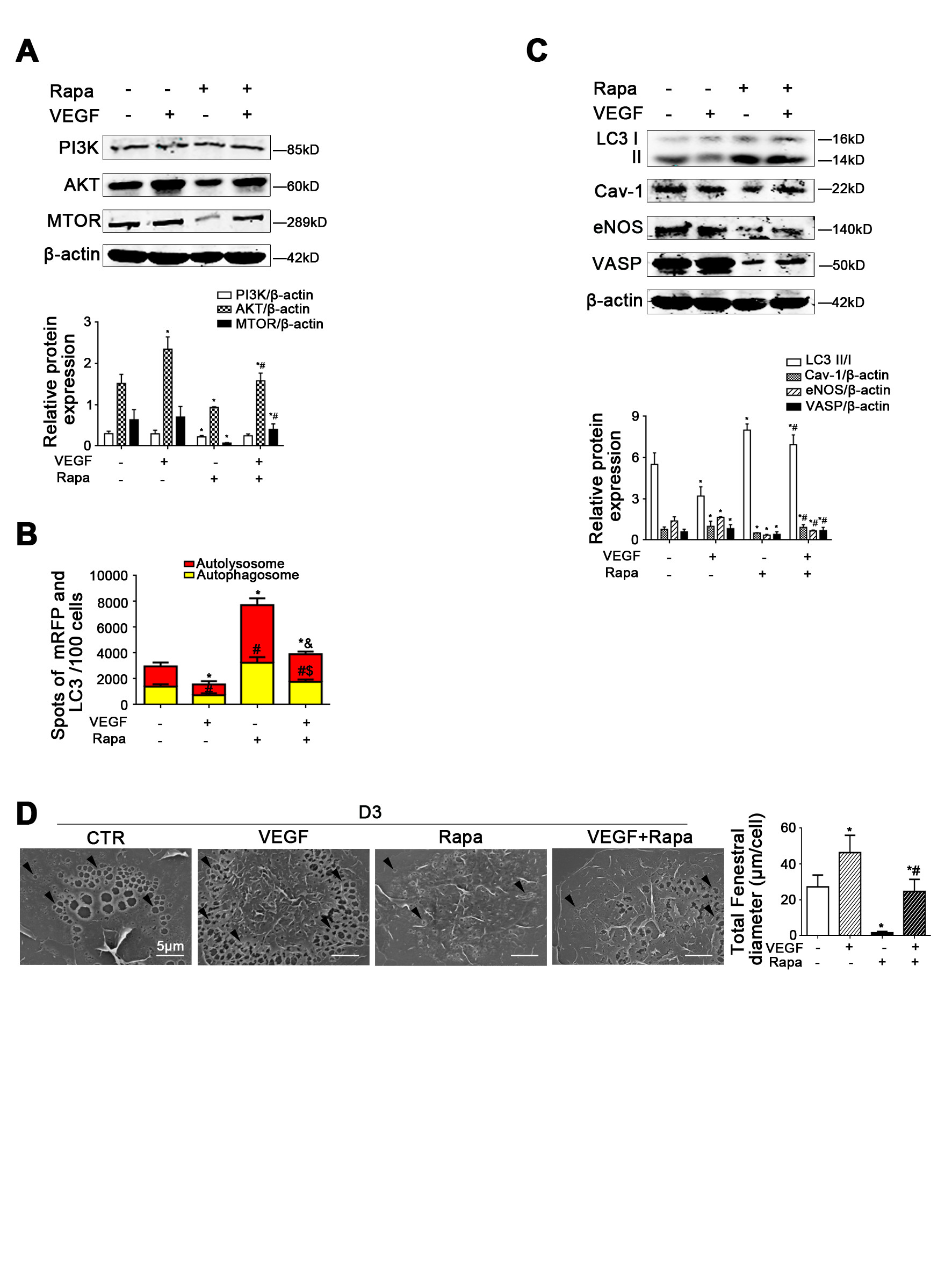


**Supplementary Figure 3. Rapamycin promoted disappearance of LSECs fenestrae maintained by VEGF via enhancing autophagic degradation of Cav-1.** Primary rat LSECs cultured *in vitro*, pre-treated with rapamycin (10 nM), were administered with VEGF (10 ng/ml) for 3 days. (**A**) Representative immunoblots of PI3K, AKT, and MTOR in LSECs. The relative protein expression is quantified in the graph below. *P<0.05 versus the control group; #P<0.05 versus the VEGF group. (**B**) Red or yellow represents autolysosomes or autophagosomes respectively, visualized by confocal microscopy. Quantification of autophagic flux (%) in 100 cells was analyzed. *P<0.05 versus the autolysosomes of the control group; #P<0.05 versus the autophagosomes of the control group; &P<0.05 versus the autolysosomes of the VEGF group; $P<0.05 versus the autophagosomes of the VEGF group. (**C**) Representative immunoblots of LC3 II/I, Cav-1, eNOS, and VASP in LSECs. The relative protein expression is quantified in the graph below. *P<0.05 versus the control group; #P<0.05 versus the VEGF group. (**D**) Magnification of SEM of LSECs, revealing the fenestrae structures (Scale bar: 5 μm), and quantification of the total fenestral diameter, right. The black triangles indicate LSECs fenestrae structures. *P<0.05 versus the control group; #P<0.05 versus the VEGF group.


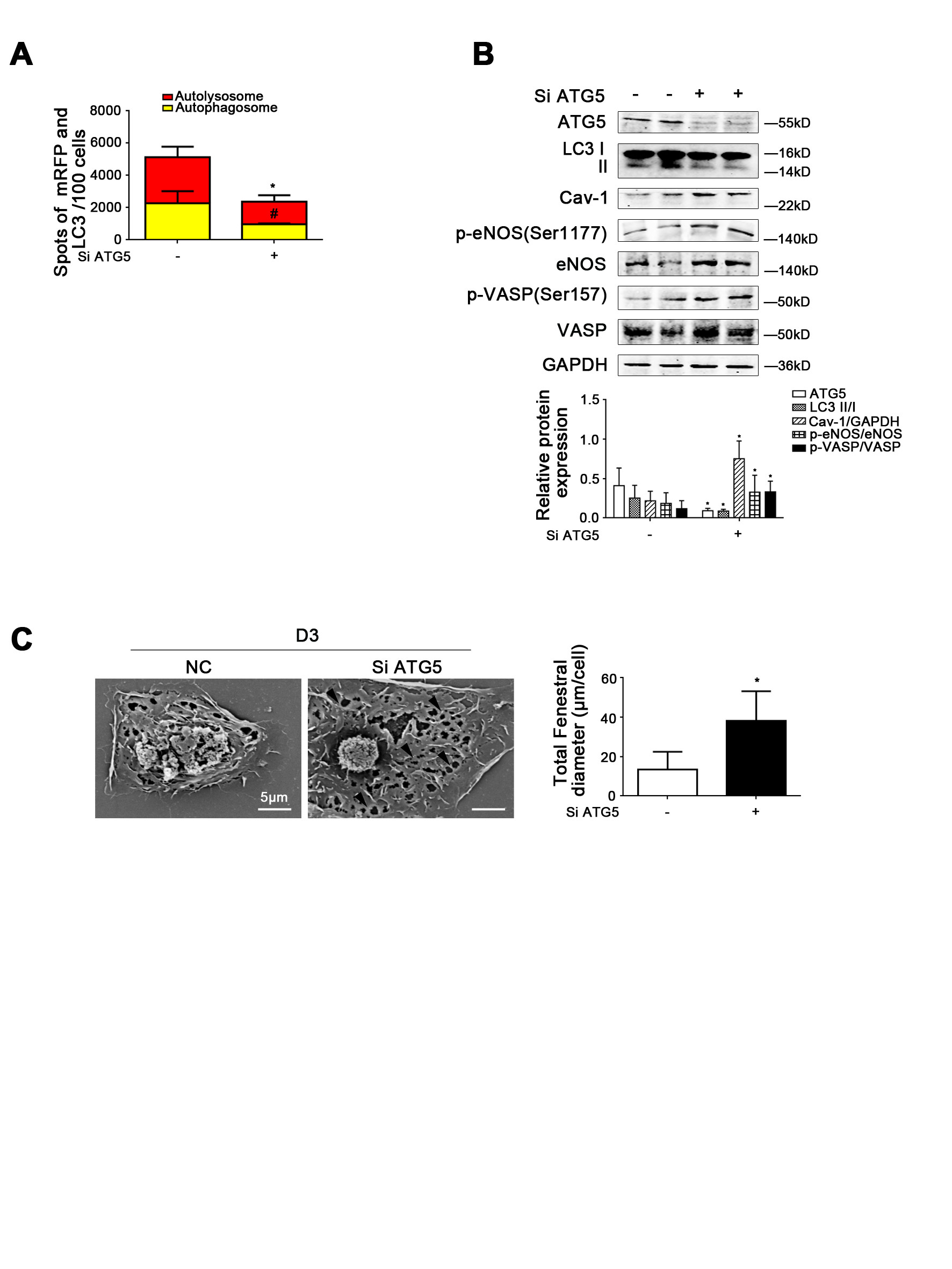


**Supplementary Figure 4. Knockdown of siATG5 maintained LSECs defenestration via reducing autophagic degradation of Cav-1.** Primary LSECs, isolated from rats, were transfected with siRNA to knockdown Cav-1 from Day 1 to Day 3. (**A**) Red or yellow represents autolysosomes or autophagosomes respectively, visualized by confocal microscopy. Quantification of autophagic flux (%) in 100 cells was analyzed. *P<0.05 versus the autolysosomes in the NC group; #P<0.05 versus the autophagosomes in the NC group. (**B**) Representative immunoblots of ATG5, LC3 II/I, Cav-1, p-eNOS (Ser1177), eNOS, p-VASP (Ser157), and VASP in LSECs analyzed by western blot. The relative protein expression is quantified in the graph, down. *P<0.05 versus the NC group. (**C**) Magnification of SEM of LSECs in the two groups (NC and siATG5) on Day 3, revealing the fenestrae structures (Scale bar: 5 μm), and quantification of the total fenestral diameter, right. The black triangles indicate LSECs fenestrae structures. *P<0.05 versus the NC group.


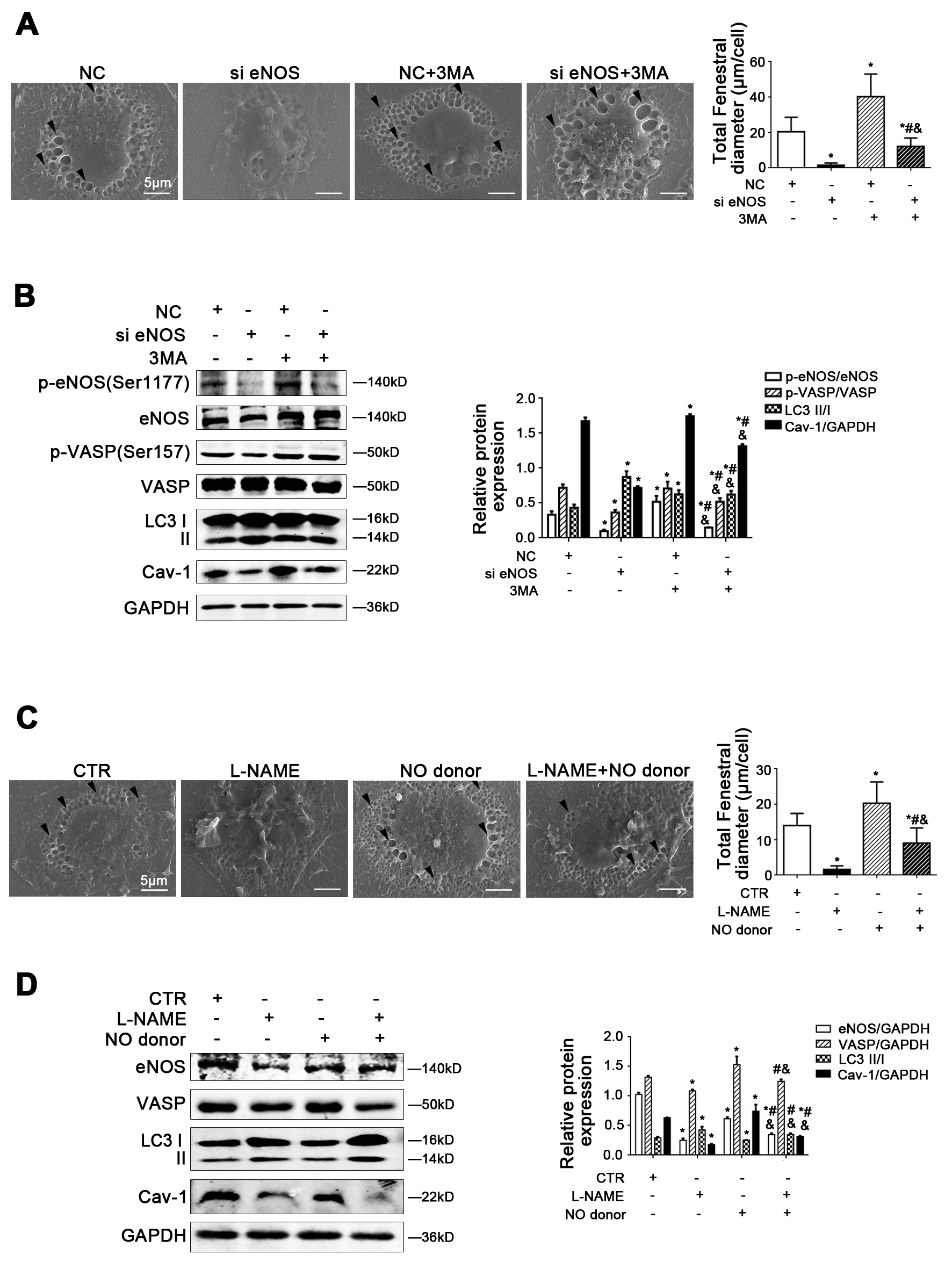


**Supplementary Figure 5. Inhibiting the NO-dependent pathway could promote LSECs defenestration via enhancing autophagic degradation of Cav-1.** Primary LSECs, isolated from normal rats, were transfected with siRNA to knockdown eNOS, followed by 3MA (10 μM)-treatment for 3 days. And primary rats LSECs cultured *in vitro*, pre-treated with L-NAME (3 mM), were administered with DETA NONOate (NO donor, 6 μM) for 3 days. (**A**) Magnification of SEM of LSECs in the four groups (NC, si eNOS, NC+3MA, si eNOS+3MA) on Day 3, revealing the fenestrae structures (Scale bar: 5 μm), and quantification of the total fenestral diameter, right. The black triangles indicate LSECs fenestrae structures. *P<0.05 versus the NC group; #P<0.05 versus the si eNOS group; &P<0.05 versus the NC+3MA group. (**B**) Representative immunoblots of p-eNOS (Ser1177), eNOS, p-VASP (Ser157), VASP, LC3 II/I, and Cav-1 in primary LSECs analyzed by western blot. The relative protein expression is quantified in the graph, down. *P<0.05 versus the NC group; #P<0.05 versus the si eNOS group; &P<0.05 versus the NC+3MA group. (**C**) Magnification of SEM of LSECs in the four groups (CTR, L-NAME, NO donor, L-NAME+NO donor) on Day 3, revealing the fenestrae structures (Scale bar: 5 μm), and quantification of the total fenestral diameter, right. The black triangles indicate LSECs fenestrae structures. *P<0.05 versus the CTR group; #P<0.05 versus the L-NAME group; &P<0.05 versus the NO donor group. (**D**) Representative immunoblots of eNOS, VASP, LC3 II/I, and Cav-1 in primary LSECs analyzed by western blot. The relative protein expression is quantified in the graph, down. *P<0.05 versus the CTR group; #P<0.05 versus the L-NAME group; &P<0.05 versus the NO donor group.


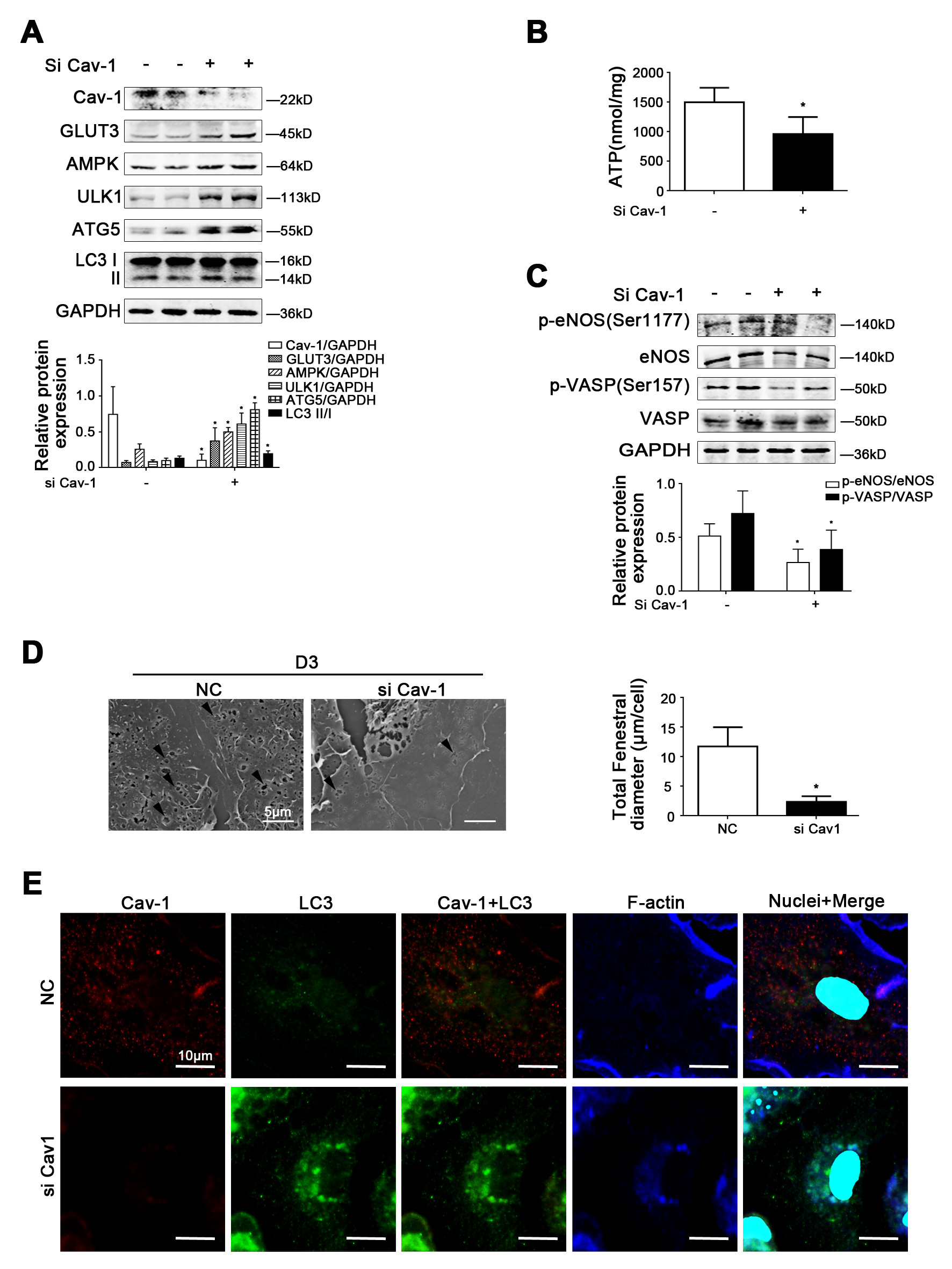


**Supplementary Figure 6. Knockdown of Cav-1 promotes LSECs defenestration via the augment of autophagy and F-actin remodeling.** Primary LSECs, isolated from normal rats, were transfected with siRNA to knockdown Cav-1. (**A**) Protein levels of Cav-1, GLUT3, AMPK, ULK1, ATG5, and LC3 II/I in primary LSECs analyzed by western blot. The relative protein expression is quantified in the graph, down. *P<0.05 versus the NC group. (**B**) The quantification of ATP level in LSECs. *P<0.05 versus the NC group. (**C**) p-eNOS (Ser1177), eNOS, p-VASP (Ser157), and VASP in primary LSECs analyzed by western blot. The relative protein expression is quantified in the graph, down. *P<0.05 versus the NC group. (**D**) Magnification of SEM of LSECs in the two groups (NC and si Cav-1) on Day 3, revealing the fenestrae structures (Scale bar: 5 μm), and quantification of the total fenestral diameter, right. The black triangles indicate LSECs fenestrae structures. *P<0.05 versus the NC group. (**E**) The co-localization of LC3 (green) with Cav-1 (red) and F-actin (blue) in LSECs of the two groups (NC and si Cav-1), shown by immunofluorescence. Scale bar: 10 μm.


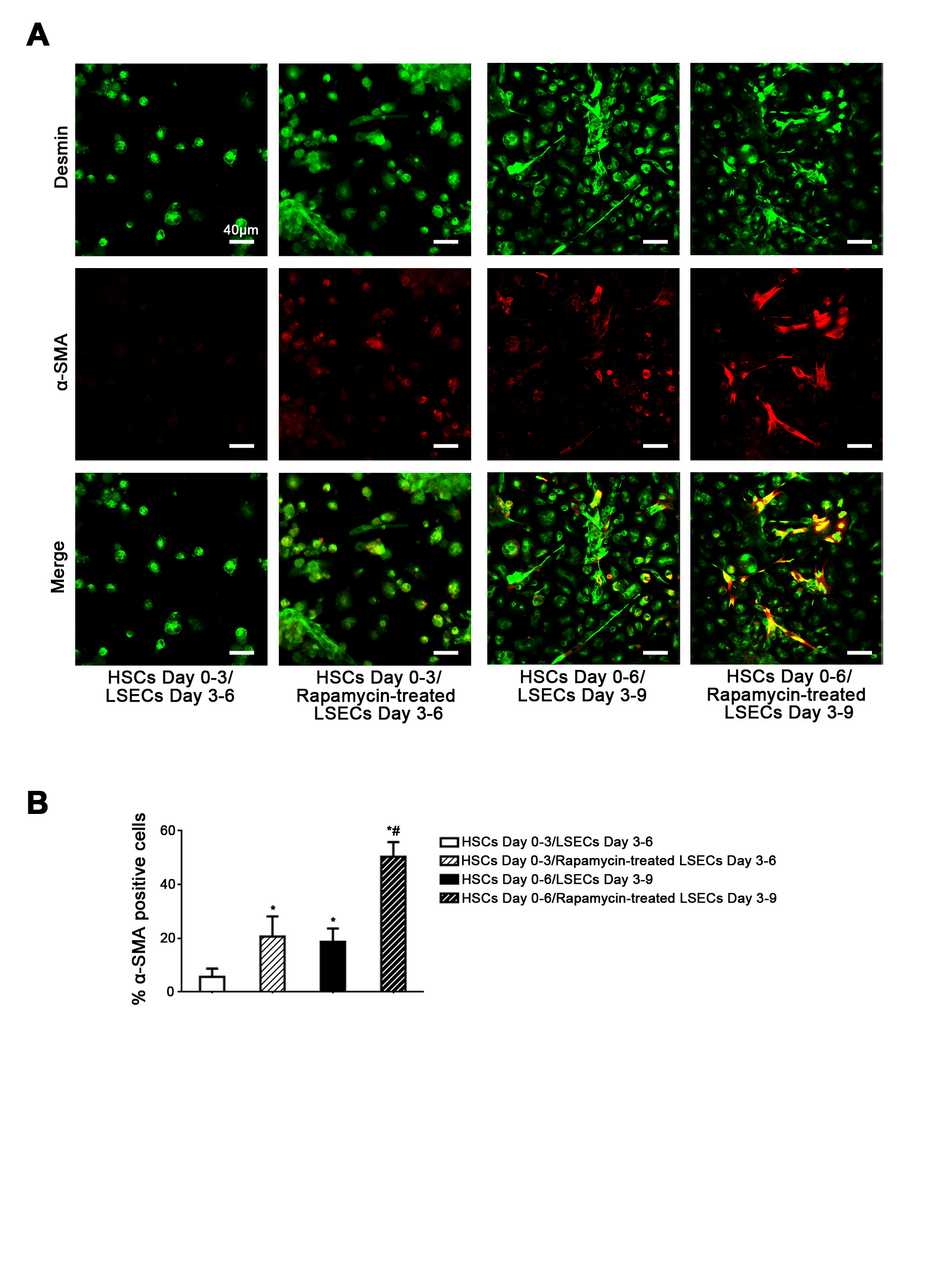


**Supplementary Figure 7. Rapamycin-induced defenestrated LSECs activated HSCs due to enhanced autophagy.** Primary rats LSECs, treated by rapamycin for 3 days, co-cultured with fresh primary HSCs isolated from normal rats for 3 days and 6 days. (**A**) Representative photomicrographs of quiescent and activated HSCs. *HSCs Day 0-3/LSECs Day 3-6 (Group 1)*: Fenestrated LSECs cultured alone from days 0 to 3, followed by co-culture with quiescent HSCs isolated from rat liver for 3 days (note: LSECs cultured from days 0 to 3 are fenestrated). *HSCs Day 0-3/Rapamycin-treated LSECs Day 3-6 (Group 2)*: LSECs were treated by rapamycin from days 0 to 3, followed by co-culture with quiescent HSCs isolated from rat liver for 3 days (note: Rapamycin promoted LSECs defenestration due to autophagy on the 3^rd^ day). *HSCs Day 0-6/LSECs Day 3-9 (Group 3)*: LSECs cultured alone from days 0 to 3, followed by co-culture with quiescent HSCs isolated from rat liver for 6 days (note: LSECs cultured alone from days 5 to 9 are defenestrated). *HSCs Day 0-6/Rapamycin-treated LSECs Day 3-9 (Group 4)*: LSECs were treated by rapamycin from days 0 to 3, followed by co-culture with quiescent HSCs isolated from rat liver for 6 days (note: Rapamycin-treated LSECs cultured from days 3 to 9 are defenestrated). Scale bar: 40 μm. Activated HSCs were labeled by α-SMA (red) and HSCs were labeled by desmin (green). (**B**) The percentage of α-SMA-positive HSCs determined by confocal microscopy: Compared with HSCs co-cultured with fenestrated LSECs for 3 and 6 days, the percentage of α-SMA-positive HSCs is higher in total HSCs cultured with rapamycin-induced defenestrated LSECs. *P<0.05 versus Group 1; #P<0.05 versus Group 3.
