# Supplementary figures and images for: Autophagic degradation of caveolin-1 promotes liver sinusoidal endothelial cells defenestration
Source: Cell Death Dis. 2018 May 14;9(5):576. doi: 10.1038/s41419-018-0567-0 (PMC5951836; doi:10.1038/s41419-018-0567-0)

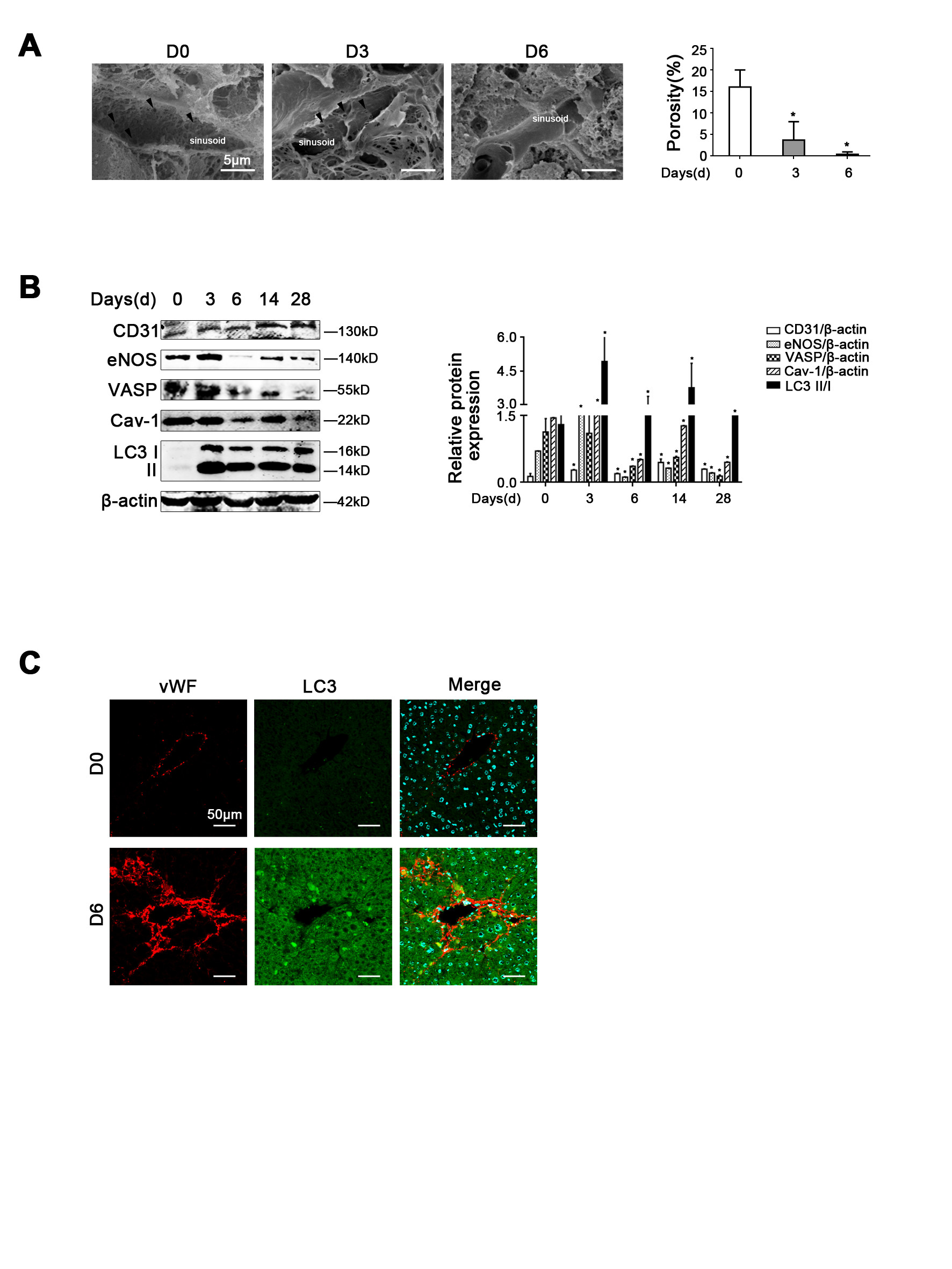

Supplement: Supplementary file 2 — Supplementary figure 1 [file 41419_2018_567_MOESM2_ESM.jpg]

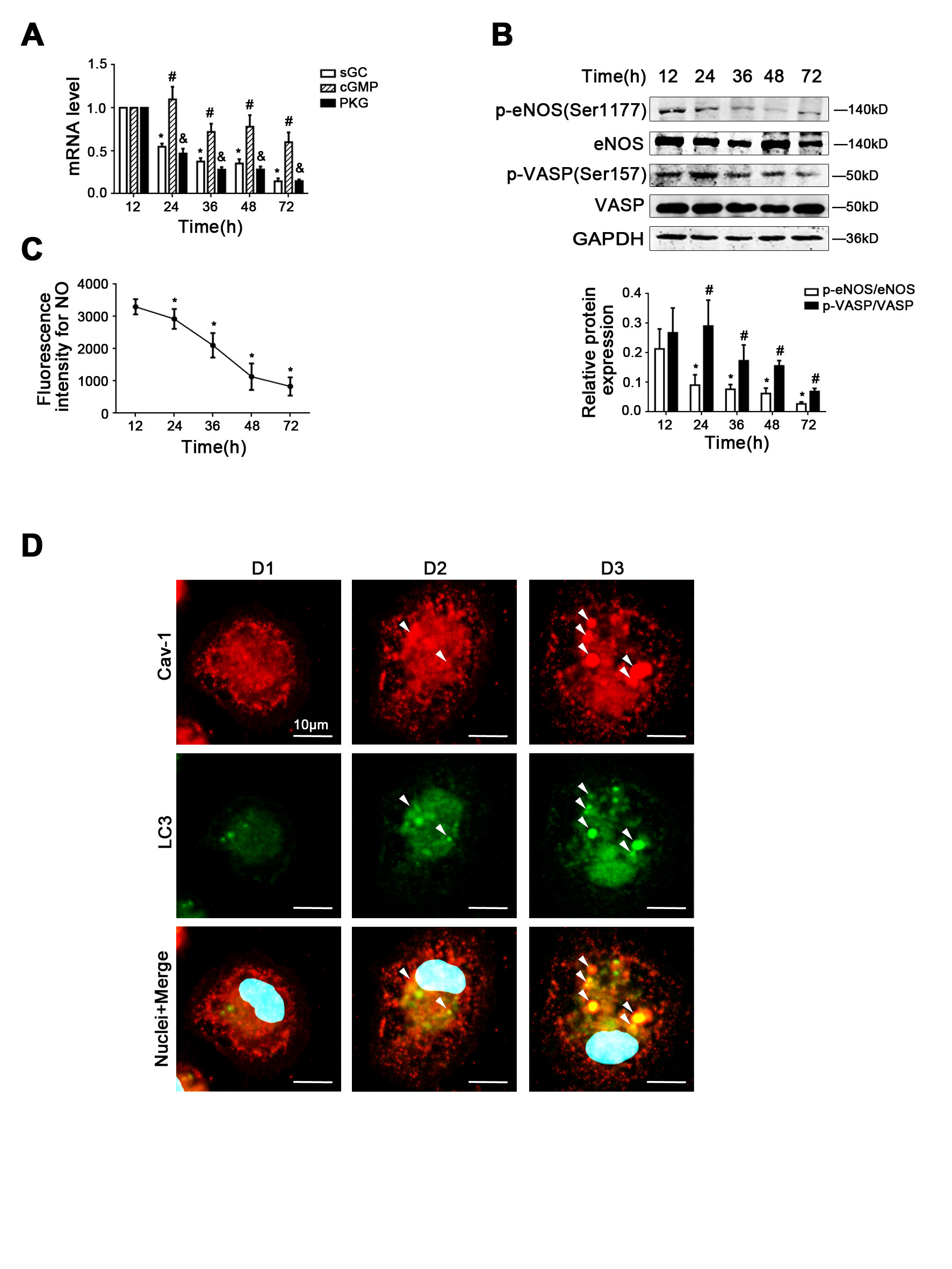

Supplement: Supplementary file 3 — Supplementary figure 2 [file 41419_2018_567_MOESM3_ESM.jpg]

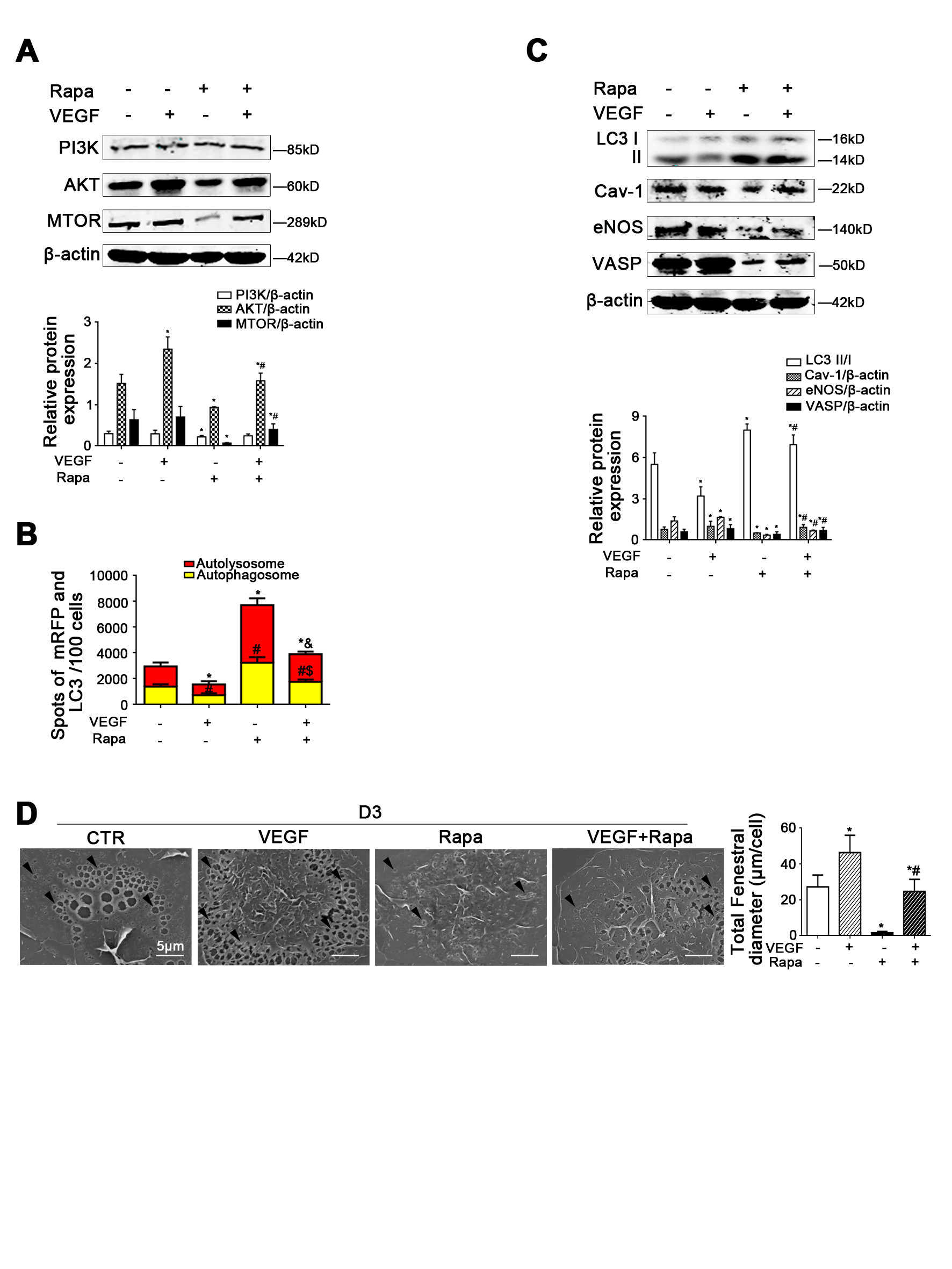

Supplement: Supplementary file 4 — Supplementary figure 3 [file 41419_2018_567_MOESM4_ESM.jpg]

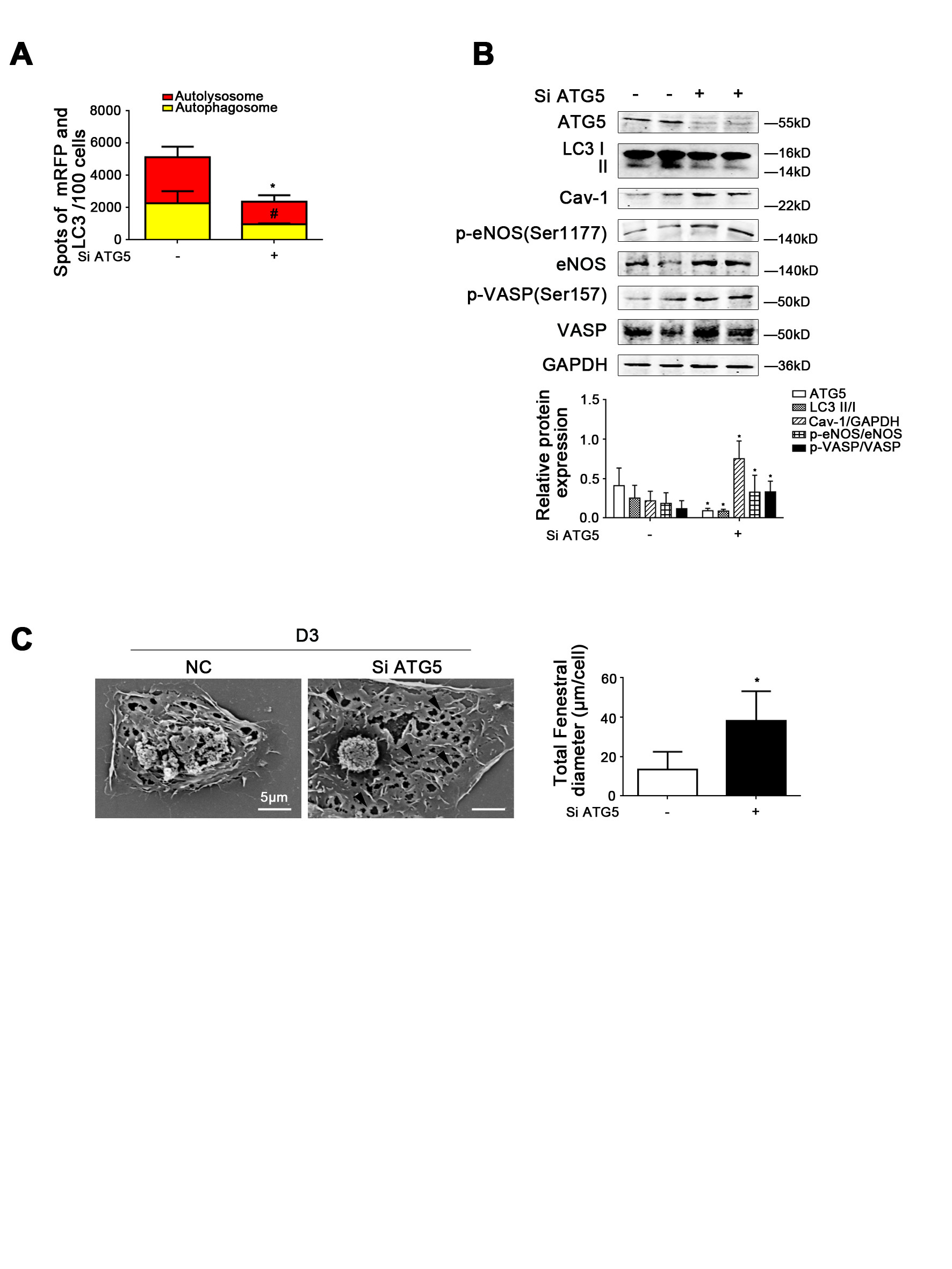

Supplement: Supplementary file 5 — Supplementary figure 4 [file 41419_2018_567_MOESM5_ESM.jpg]

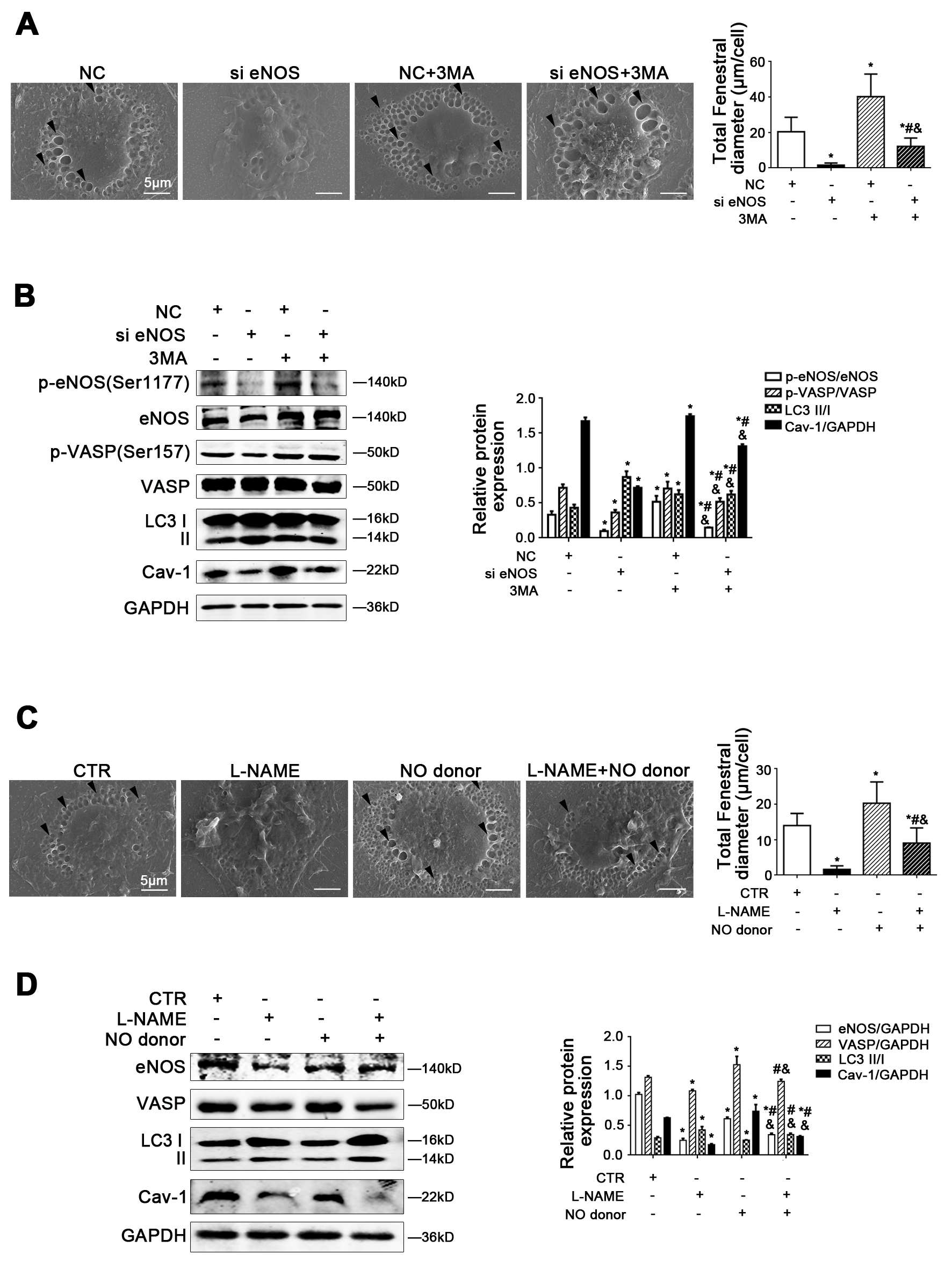

Supplement: Supplementary file 6 — Supplementary figure 5 [file 41419_2018_567_MOESM6_ESM.jpg]

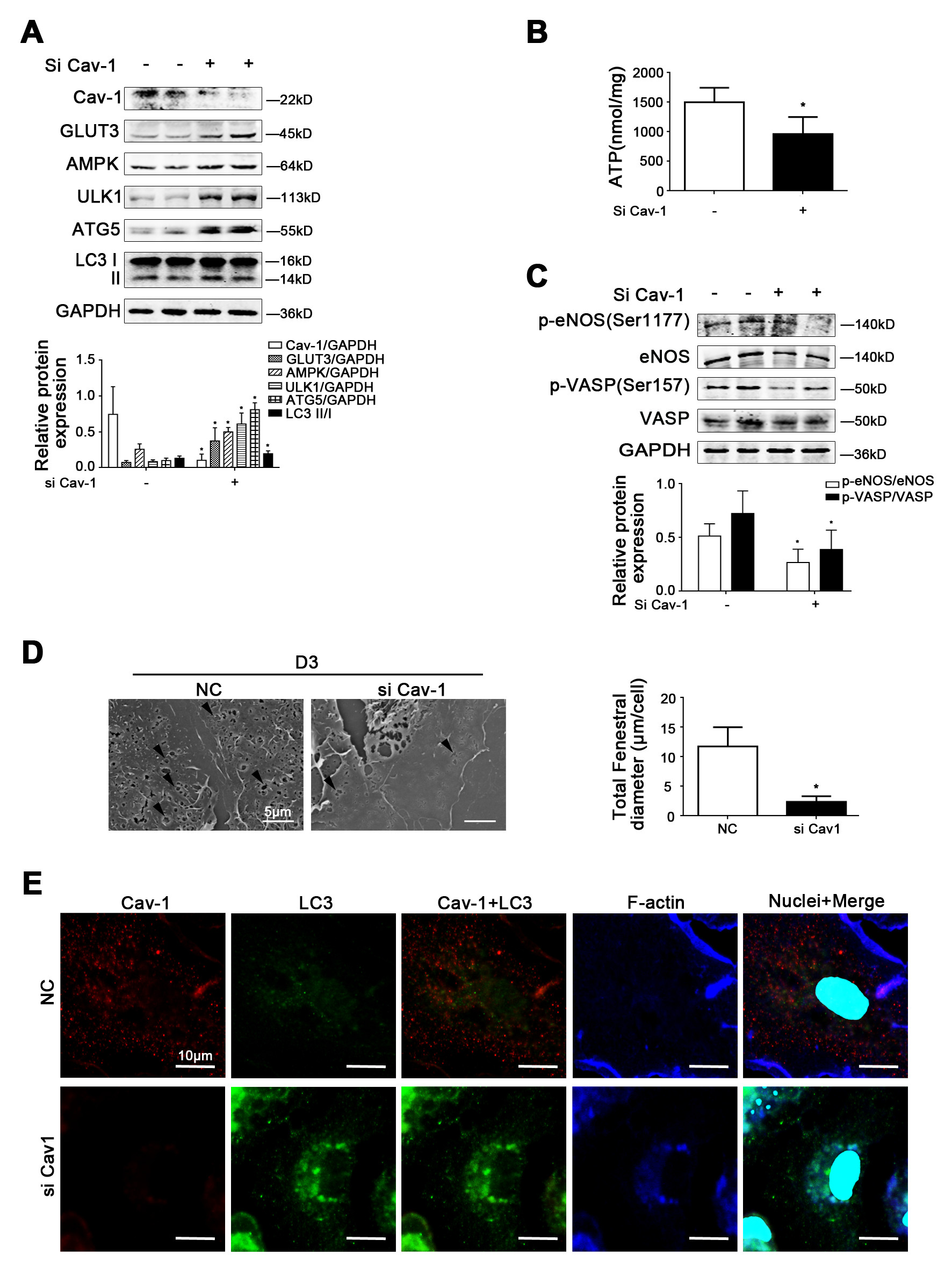

Supplement: Supplementary file 7 — Supplementary figure 6 [file 41419_2018_567_MOESM7_ESM.jpg]

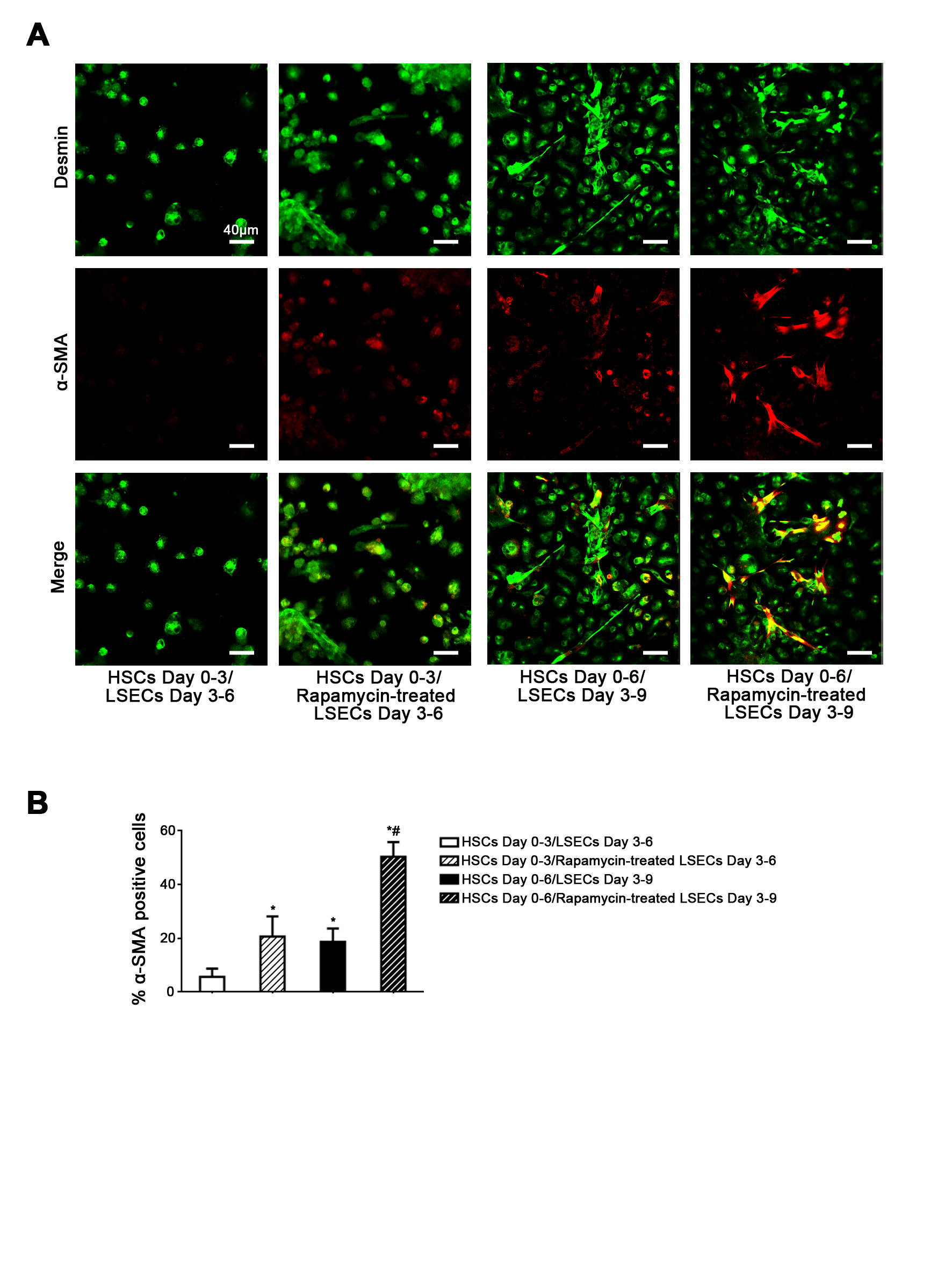

Supplement: Supplementary file 8 — Supplementary figure 7 [file 41419_2018_567_MOESM8_ESM.jpg]
